# Pistillody mutant reveals key insights into stamen and pistil development in wheat (*Triticum aestivum* L.)

**DOI:** 10.1186/s12864-015-1453-0

**Published:** 2015-03-19

**Authors:** Zaijun Yang, Zhengsong Peng, Shuhong Wei, Mingli Liao, Yan Yu, Zeyan Jang

**Affiliations:** Key Laboratory of Southwest China Wildlife Resources Conservation, College of Life Science, China West Normal University, Nanchong, 637009, Sichuan China

**Keywords:** Pistillody, Pistil, Stamen, RNA sequencing, Wheat

## Abstract

**Background:**

The pistillody mutant wheat (*Triticum aestivum* L.) plant HTS-1 exhibits homeotic transformation of stamens into pistils or pistil-like structures. Unlike common wheat varieties, HTS-1 produces three to six pistils per floret, potentially increasing the yield. Thus, HTS-1 is highly valuable in the study of floral development in wheat. In this study, we conducted RNA sequencing of the transcriptomes of the pistillody stamen (PS) and the pistil (P) from HTS-1 plants, and the stamen (S) from the non-pistillody control variety Chinese Spring TP to gain insights into pistil and stamen development in wheat.

**Results:**

Approximately 40 Gb of processed reads were obtained from PS, P, and S. *De novo* assembly yielded 121,210 putative unigenes, with a mean length of 695 bp. Among these high-quality unigenes, 59,199 (48.84%) had at least one significant match with an existing gene model. A total of 23, 263, and 553 differentially expressed genes were identified in PS *vs*. P, PS *vs*. S, and P vs. S, respectively, with differences in expression greater than five-fold. Among the differentially expressed genes, 206 were highly correlated with stamen and pistil development. These genes include *WM27B*, *DL*, *YAB1*, *YABBY4*, *WM 5*, *CER 1*, and *WBLH1*, which have been implicated in flower development. The expression patterns of 25 differentially expressed genes were confirmed through quantitative real-time reverse transcription PCR.

**Conclusions:**

Analysis of this transcriptome resource enabled us to characterize gene expression profiles, examine differential gene expression, and produce a candidate gene list related to wheat stamen and pistil development. This work is significant for the development of genomic resources for wheat, and provides important insights into the molecular mechanisms of wheat stamen and pistil development.

**Electronic supplementary material:**

The online version of this article (doi:10.1186/s12864-015-1453-0) contains supplementary material, which is available to authorized users.

## Background

Wheat (*Triticum aestivum* L.) is a major staple food crop in several parts of the world, in terms of its cultivation area and use as a food source. Increasing yield to meet the increasing global demand for the crop is the main goal of wheat production. One way to improve the wheat yield potential is to increase the grain number per spike and unit area [1,2]. For this purpose, wheat scientists have considered a wide range of genetic variations in the morphological structure of wheat to obtain high grain numbers per spike. These morphological variations include supernumerary spikelets, multi-spikelet [3], and multi-row spikes [4]. Peng [5] selected a three-pistil (TP) mutant with normal spike morphology that produced three pistils per floret. Consequently, a floret could develop into three seeds, thereby increasing the seed number per spike. Meanwhile, the novel pistillody mutant, HTS-1, was screened from Chinese Spring TP (CSTP), which is a near-isogenic line of the common wheat variety Chinese Spring with the *Pis1* gene derived from the TP mutant [6]. HTS-1 plants exhibit a novel phenotype that transforms all or parts of the stamen into pistils or pistil-like structures. In recent years, the alloplasmic lines N26 [7] and (cr)-CSdt7BS [8] have been used to determine the genetic and molecular mechanisms of wheat pistillody [9-12]. Nuclear-cytoplasm interaction [8,12] causes pistillody in N26 and (cr)-CSdt7BS. However, pistillody in HTS-1 is caused by the interaction of the recessive karyogenes *hts1* and *hts2* [6]. Therefore, HTS-1 is genetically different from the previously reported lines (cr)-CSdt7BS and N26. Wheat florets are considered extremely stable and have a few reported mutants. Previous studies on floret mutants only provided a superficial understanding of floral organ identity determination in wheat plants. Consequently, HTS-1 is a significant genetic material to study the floral development of wheat; this line also has the potential to increase wheat yield.

Compared with studies on the functions of single or few genes during flower development [13,14], the underlying genetic determinants that control flower development have received relatively little attention in wheat. Moreover, the genes and their corresponding expression patterns related to pistil and stamen development have yet to be reported. Previous studies on expressed sequence tag sequences generated a large number of cDNA sequences for the wheat TriFLDB database (http://trifldb.psc.riken.jp/index.pl), which contains approximately 16,000 full-length cDNAs [15]. Traditional sequencing methods have been used on randomly selected cDNA clones from various tissues; however, these methods obtained a low coverage of less-abundant or rare transcripts, which usually have vital functions. A novel approach to transcriptome profiling, called RNA sequencing (RNA-seq) has been developed recently, this method is based on next-generation sequencing (NGS) technologies [16,17]. RNA-seq has been widely applied in plant biology, particularly in model species, such as *Arabidopsis* [18], and crop plants, such as rice [19], maize [20], and wheat [21].

In the present study, we used RNA-seq to investigate and compare the transcriptomes of pistillody stamen (PS) and the pistil (P) from HTS-1 plants, and of the stamen (S) from the non-pistillody control variety CSTP. The results of this study provide insights into P and S development in wheat.

## Results

### Comparison of the morphological structures of PS, P, and S

**Figure 1:**
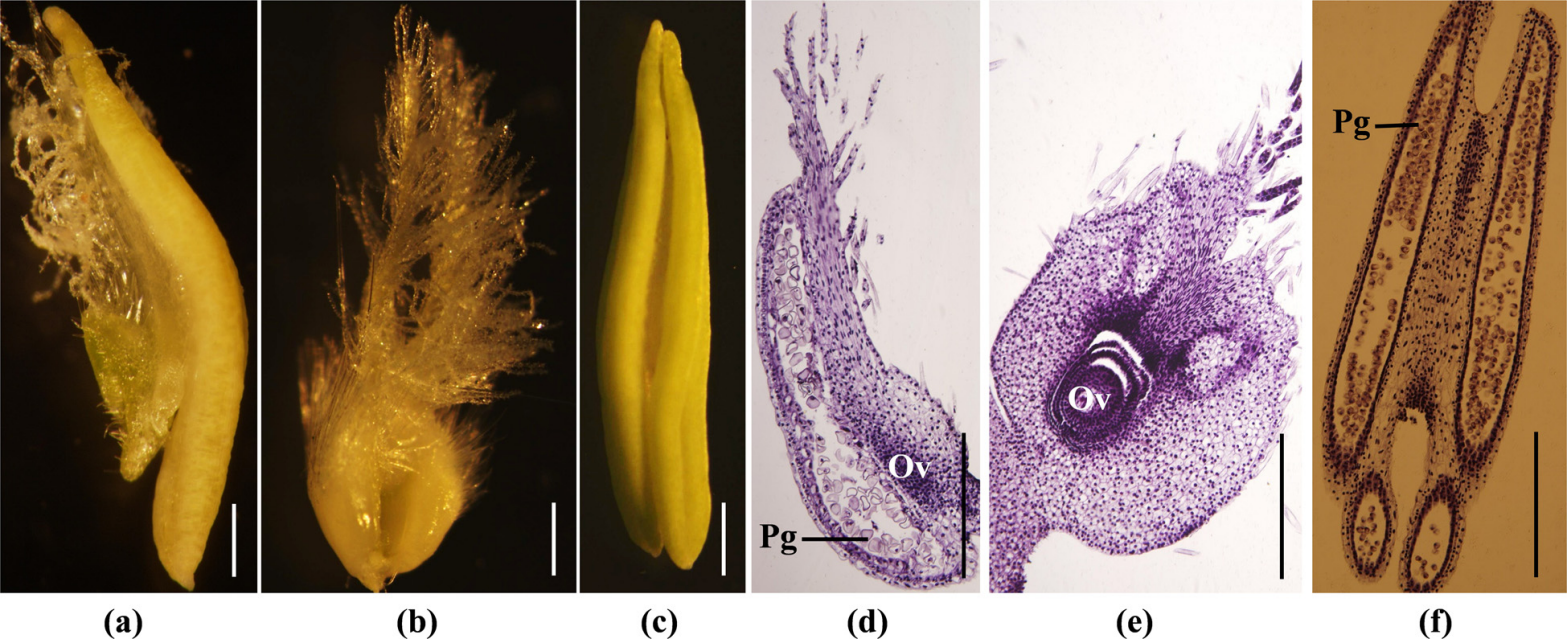
**Comparison of morphological structure of pistillody stamen, pistil and stamen (a) pistilldoy stamen (b) normal pistil (c) normal stamen (d) longitudinal section of pistillody stamen (e) longitudinal section of normal pistil (f) longitudinal section of normal stamen.** Ov: ovule, Pg: pollen grain. Bars = 1 mm.

Peng et al. [6] observed pistillody in HTS-1. HTS-1 is a CSTP sib-line that carries the *Pis1* gene. However, HTS-1 plants exhibit different florets; i.e., some HTS-1 stamens turn into pistils or a combination of stamens and pistils. As shown in Figure 1-a, the anther-like structure bears a tuft of ‘stigma hair’ at the right. A normal pistil and stamen are shown in Figure 1-b and 1-c. To compare the structures of PS, P, and S, each part was sectioned longitudinally and examined for histological modifications. The P showed well-developed ovules (Figure 1-e) and S contained normal pollen grains (Figure 1-f). PS (partially transformed stamen) contained ovule-like structures and had a pistil-like form; however, the ovules were underdeveloped and sometimes contained deformed pollen grains (Figure 1-d).

### Transcriptome sequencing and *de novo* assembly

Three cDNA libraries (PS, P, and S; Figure 1) were constructed with the respective total RNA from PS, P, and S., The prepared libraries were sequenced on an Illumina Hiseq 2000 platform. A total of 134,561,846, 122,116,204, and 152,071,674 bp paired-end reads were obtained for PS, P, and S, respectively, which corresponded to a total size of 40.88 Gbp after low-quality reads and adapter sequences were removed (Table 1). All paired-end reads were pooled together and assembled *de novo* using Trinity (v2012-10-05) [22]. Finally, we obtained 330,912 transcripts with lengths ≥200 bp. The mean length of these transcripts was 1,071 bp, and the maximum length was 20,603 bp (Table 1). The transcripts were assembled into 121,210 putative unigenes. The sequence information of all Illumina reads was deposited in the National Center for Biotechnology Information (NCBI) under the accession number SRP038912. The mean length of the putative unigenes was 695 bp. Among all the putative unigenes, 23,137 were longer than 1000 bp, which represented 19.1% of the total (Table 1). The size distribution of the assembly transcripts and unigenes is shown in Additional file 1: Figure S1. The 121,210 putative unigenes were aligned against the draft sequence of the bread wheat genome (IWGSP 1.21); 100,401 unigenes (82.2%) could be mapped to the exon regions.

**Table 1:** **Summary of RNA-seq and de novo assembly of** ***T. aestivum*** **unigenes**

Number of clean reads	408749724
Number of ≥200 bp transcripts	330912
Number of ≥2000 bp transcripts	48158
Number of ≥1000 bp transcripts	131321
Mean length of transcripts (bp)	1071
Max length of transcripts (bp)	20603
N50 length of transcripts (bp)	1684
Number of Unigenes	121210
Number of ≥2000 bp Unigenes	8553
Number of ≥1000 bp Unigenes	23137
Mean length of Unigenes (bp)	695
Max length of Unigenes (bp)	20603
N50 length of Unigenes (bp)	1174

### Functional annotation of the unigenes

The entire set of unigenes was annotated on the basis of their similarities with known or putative annotations in public databases, including GenBank NR, GenBank NT, KO, SwissProt, PFAM, GO, and KOG (E values ≤1e − 5 for GenBank NR, GenBank NT, and SwissProt; E values ≤1e − 3 for KOG [23]). Among the 121,210 high-quality unique sequences, only 59,199 (48.84%) had at least one significant match with an existing gene model in BLAST searches (Table 2).

**Table 2:** **Summary statistics of functional annotation for** ***T. aestivum*** **unigenes in public protein databases**

	**No. of unigene hits**	**Percentage (%)**
Annotated in NR	42865	35.36
Annotated in NT	39521	32.6
Annotated in KO	5491	4.53
Annotated in SwissProt	24130	19.9
Annotated in PFAM	30735	25.35
Annotated in GO	35481	29.27
Annotated in KOG	12087	9.97
Annotated in all Databases	2519	2.07
Annotated in at least one Database	59199	48.84
Total Unigenes	121210	100

**Figure 2:**
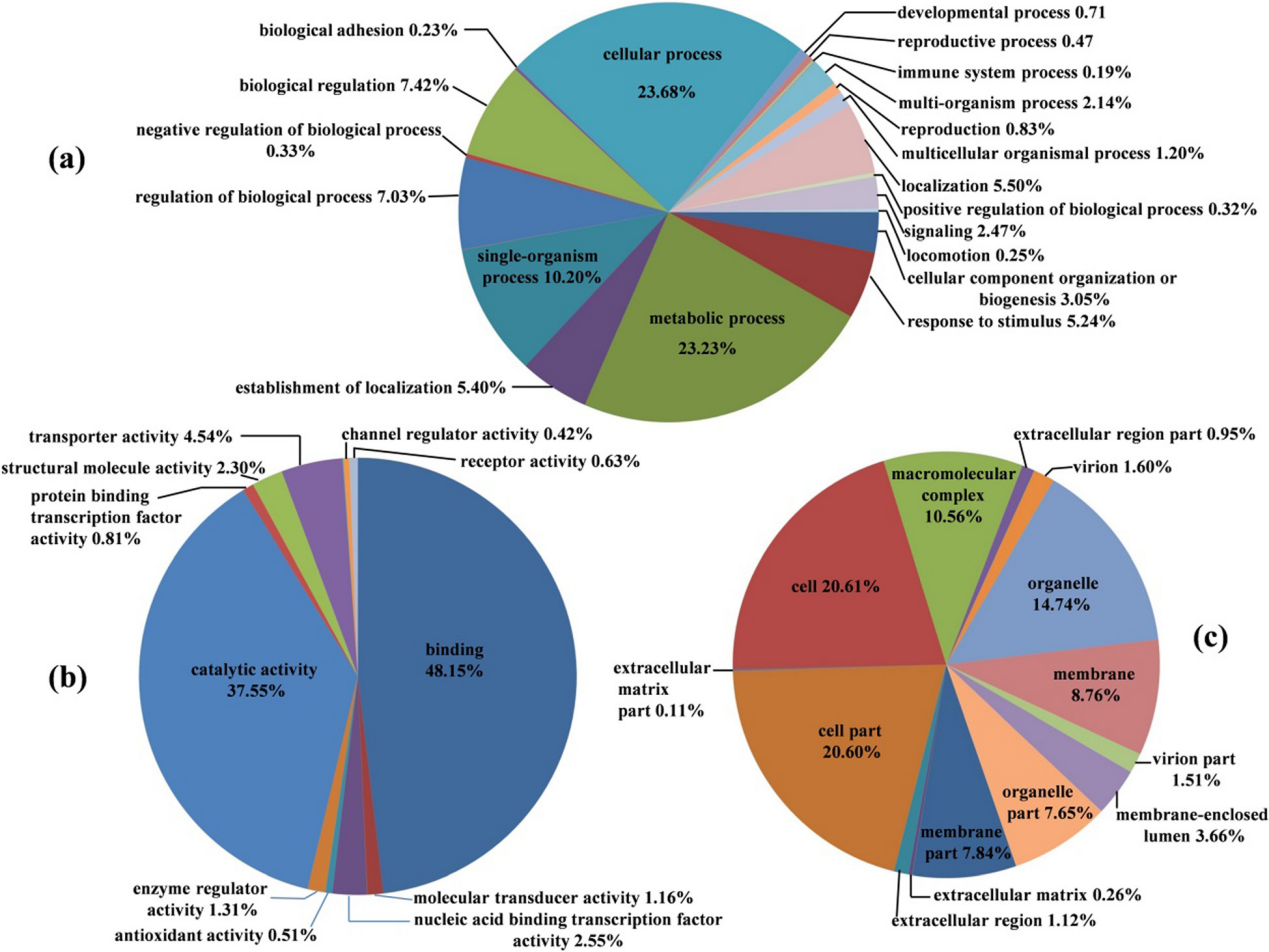
**Gene Ontology (GO) classification of unigenes derived from pistilldoy stamen, pistil and stamen in wheat (a) biological processes (b) molecular function (c) cellular components.**

Gene ontology (GO) was employed to identify the functional categories of the annotated unigenes and to classify the transcripts with known annotated proteins. 35,481 unigenes (29.27%) had significant similarities to Nr and Pfam proteins and were assigned under GO terms. In several cases, multiple terms were assigned to the same transcript. The analysis produced 21,037 assignments to biological processes, 14,548 to cellular components, and 11,022 to molecular functions (Figure 2). Most of the biological process categories were related to metabolic processes (GO: 0008152, 23.23%) and cellular processes (GO: 0009987, 23.68%). Under the category of cellular components, 20.60% and 20.61% of the unigenes were located in cell parts (GO: 0044464) and cells (GO: 0005623), respectively. Among the molecular functions, the majority of the GO terms were grouped into either binding (GO: 0005488, 48.15%) or catalytic activity (GO: 0008152, 37.55%).

To further understand the biological functions and interactions of the gene products, a pathway-based analysis was conducted using the *Kyoto Encyclopedia of Genes and Genomes* (KEGG) pathway database. KEGG records the molecular interaction networks in cells with variants that are specific to particular organisms. Unigenes with annotated Nr and Pfam proteins were mapped to the KO database using KOBAS (KEGG Orthology-Based Annotation System, v2.0) [24]. The results showed that 11,848 unigenes could be assigned to 243 KEGG pathways for PS, P, and S transcriptomes (Additional file 2: Table S1). Most unigenes of the 243 KEGG pathways were ribosome pathways (324 genes). Glucosinolate biosynthesis, peptidoglycan biosynthesis, geraniol degradation, and polyketide sugar unit biosynthesis had the least number of unigenes (one gene).

### Differentially expressed genes (DEGs) among PS, P, and S

**Figure 3:**
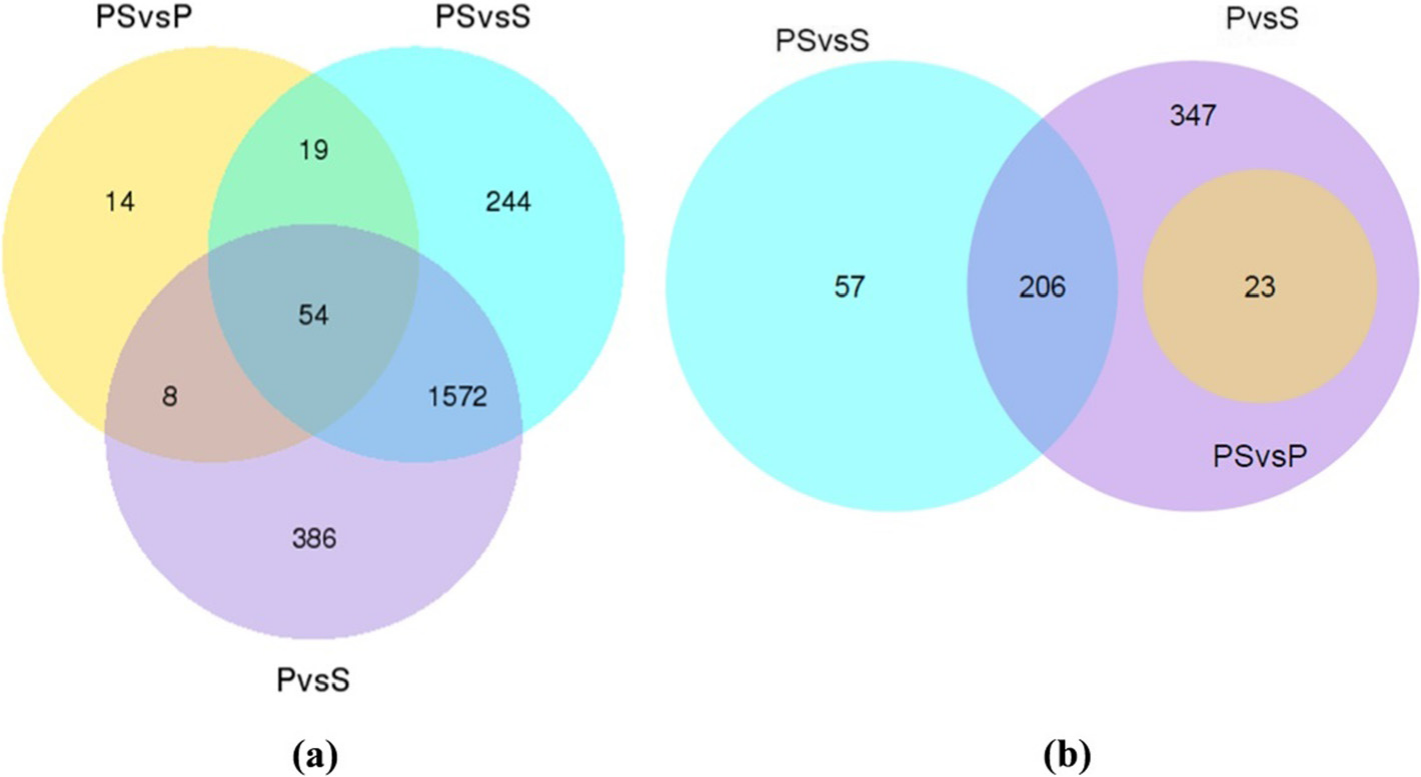
**Venn diagram showing differentially expressed genes among pistillody stamen, pistil and stamen (a) transcripts detected with |log2 fold change| > 2 (b) transcripts detected with |log2 fold change| > 5.**

The tag frequency differences that appeared in the PS, P, and S libraries were used to estimate the gene expression levels that corresponded to stamen and pistil development (0.3 reads per kilobase of transcript per million reads mapped was used as an expression threshold that was well above the background). The numbers of DEGs in PS *vs*. P, PS *vs*. S, and P *vs*. S were 95, 1,889 and 2,020, respectively, for transcripts detected with |log2 fold change| > 2 (Figure 3a). A total of 4,004 unigenes were identified as DEGs in at least two libraries. Among these shared DEGs, 1,626 common DEGs were found between PS *vs*. S and P *vs*. S, which may contribute to stamen and pistil development. The DEGs with |log2 fold change| > 5 are shown in Figure 3b; at this level, PS *vs*. P, PS *vs*. S, and P *vs*. S had 23, 263, and 553 DEGs, respectively. Among the DEGs within this threshold, 206 DEGs were common in PS *vs*. S and P *vs*. S, including 83 upregulated genes and 123 downregulated genes (Additional file 3: Table S2). These 206 genes may have a high correlation with stamen and pistil development. PS *vs*. P had the least DEGs for |log2 fold change| > 2 or |log2 fold change| > 5, with only 95 and 23 DEGs, respectively. This trend suggested that the PS and P have similar transcript profiles.

### Functional annotation analysis of DEGs

**Figure 4:**
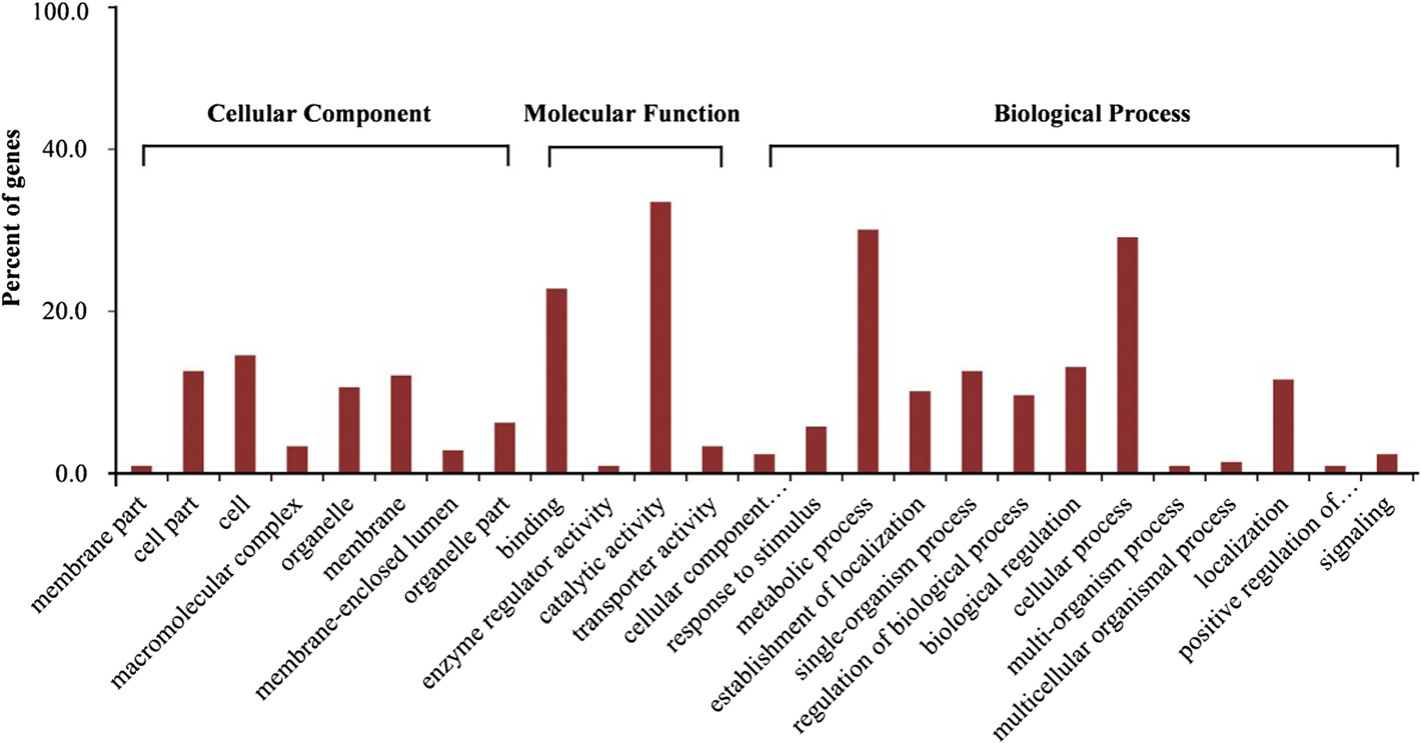
**Gene Ontology (GO) annotations of common differentially expressed genes between PS vs S and P vs S.**

The set of 206 common DEGs between PS *vs*. S and P *vs*. S was mapped in accordance with the GO biological process database to understand the functions of these DEGs; the gene expression profiles were compared with the whole transcriptome background to identify the genes involved in important biological processes. After GO Term Enrichment Analysis, 141 genes among the common DEGs were assigned to at least one term in the GO cellular component, molecular function, and biological process categories; these terms included 57 upregulated genes and 84 downregulated genes. Transcripts from the three categories were further classified into 25 functional groups, which provided an overview of the ontology of each transcriptome (Figure 4). Among the 25 functional groups, catalytic activity (33.5%), metabolic process (30.1%), and cellular process (29.1%) were prominently represented. This result indicated that the three functional groups might be related to pistil and stamen development. At least seven unigenes with high percentage identity to known flower development genes from wheat, barley, and *Brachypodium distachyon* (Table 3), were identified by comparing the DEGs found in this study with the NCBI databases.

**Table 3:** **List of previously reported genes to contribute to flower development**

**Unigene ID**	**Size (bp)**	**BLAST matching accession No.**	**Gene description**	**Sequence identity (%)**	**E value**
comp108229_c0	1098	AM502895.1	MIKC-type MADS-box transcription factor *WM27B* [*Triticum aestivum*]	98	0
comp110066_c0	1385	AB470269.1	*DL* related protein [*Triticum aestivum*]	99	0
comp110106_c0	1718	AY330228.1	*YABBY* protein (*YAB1*) [*Triticum aestivum*]	99	0
comp112313_c0	1680	XM_003569922.1	protein *YABBY 4*-like [*Brachypodium distachyon*]	90	0
comp113007_c0	1600	AY792974.1	wheat meiosis 5 (*WM5*) [*Triticum aestivum*]	97	0
comp112668_c1	1114	DQ493457.1	putative aldehyde decarbonylase enzyme *CER1* [*Hordeum vulgare*]	90	0
comp122918_c0	3622	AB546642.1	*BEL1*-type homeodomain protein *WBLH1*[*Triticum aestivum*]	99	0

### Quantitative real-time reverse transcription PCR (qRT-PCR) analysis

**Figure 5:**
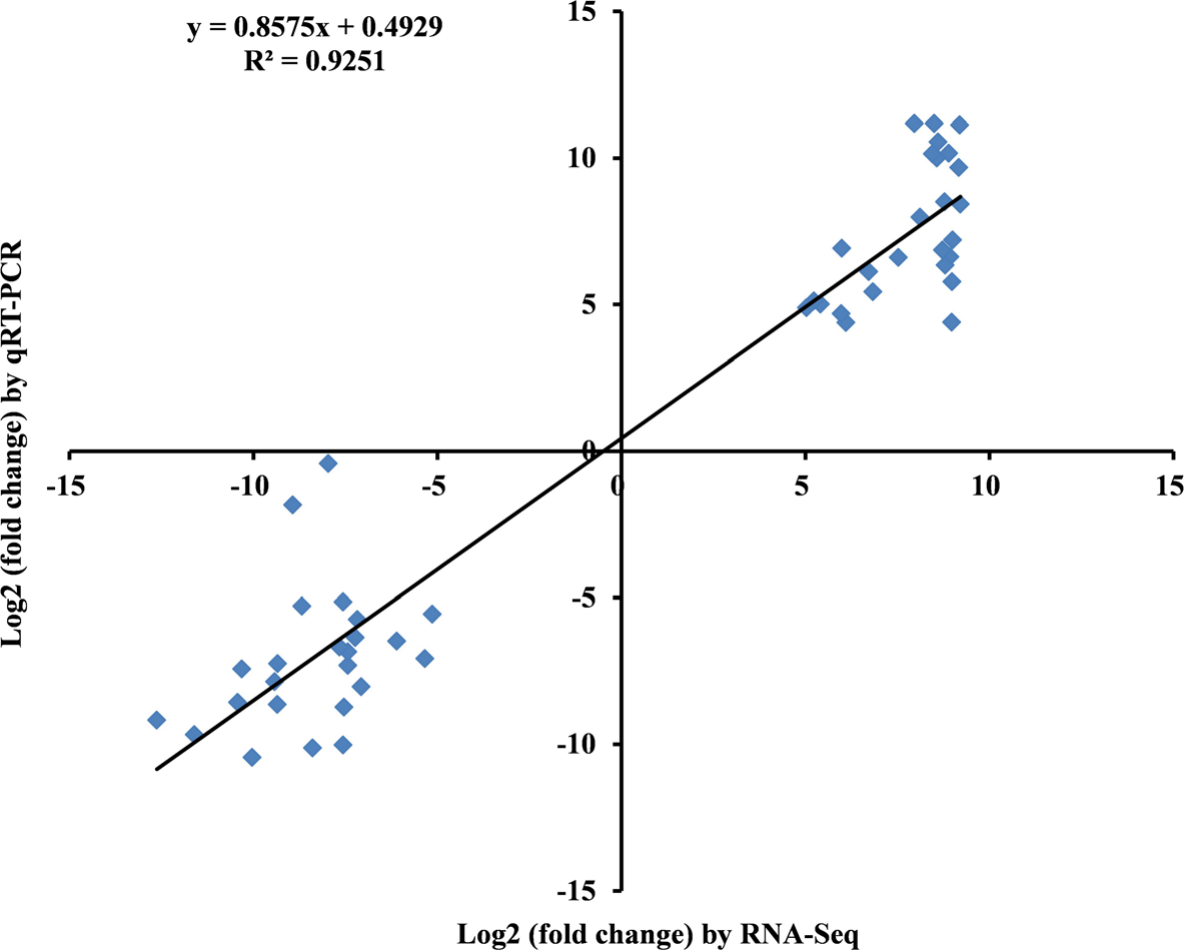
**Comparison of expression levels measured by RNA-Seq and qRT-PCR for selected 25 transcripts from 206 common differentially expressed genes.**

qRT-PCR was performed on 25 unigenes, including 13 upregulated genes and 12 downregulated genes, to validate the results of expression profiling obtained from RNA-seq. Among the 25 unigenes, 18 unigenes were randomly selected from the 206 common DEGs, and the other seven unigenes were previously reported to influence flower development namely, MIKC-type MADS-box transcription factor *WM27B* (*TaAG-4*), a *DL* related gene, *YAB1*, *YABBY 4*, *WM5*, *CER1*, and *WBLH1*. We compared the results obtained from qRT-PCR with those generated from the RNA-seq analysis of the transcripts. The trends of expression were consistent for all transcripts in both analyses, with a correlation coefficient of R^2^ = 0.9251 (Figure 5). The expression profiles of *WM27B*, *DL*, *YAB1*, *YABBY 4*, *WM5*, *CER1*, *and WBLH1* are listed in Table 4. The five genes, *WM27B*, *DL*, *YAB1*, *YABBY 4,* and *WM5*, were indeed expressed at higher levels in PS and P compared with S (26 ~ 2339-fold changes). *CER1* and *WBLH1* were downregulated genes in PS and S, and the transcript levels in S were 47 ~ 1038-fold higher than in PS and P.

**Table 4:** **The expression profiling of previously reported genes to contribute to flower development**

**Unigene ID**	**Gene name**	**Mean relative expression**			**fold changes**	
		**PS**	**P**	**S**	**PS vs. S**	**P vs. S**
comp108229_c0	WM27B	0.64	1.33	0.03	21	44
comp110066_c0	DL	1.38	1.37	0.00059	2339	2322
comp110106_c0	YAB1	1.53	1.23	0.013	118	95
comp112313_c0	YABBY4	1.18	0.93	0.036	33	26
comp113007_c0	WM5	1.78	1.52	0.051	35	30
comp112668_c0	CER1	0.0013	0.0034	1.35	1/1038	1/397
comp122918_c0	WBLH1	0.035	0.012	1.63	1/47	1/136

## Discussion

Low-cost and high-throughput NGS technologies, particularly RNA-seq, have become useful, not only for *de novo* genome assembly, molecular marker development, and genome diversity studies, but also to discover novel genes and to investigate gene expression profiles [16]. In model species, transcriptome profiling and gene expression quantification are generally performed by mapping reads from the NGS analysis to a reference genome sequence and then annotating the selected genes. The strategies for model species are not feasible in wheat because the reference genome sequence and gene annotation of wheat remain incomplete. However, an international project to achieve these goals is currently under way (International Wheat Genome Sequence Consortium, http://www.wheatgenome.org/). This project may take a considerable time because of the difficulties involved in sequencing the large (40 times larger than rice), highly repetitive, hexaploid genome of wheat. For this reason, biological analyses of wheat based on NGS data are challenging. The present study used NGS technology for wheat RNA-seq to characterize and compare the gene expression profiles among PS, P, and S to identify candidate genes related to pistil and stamen development.

In this study, Illumina sequencing from wheat PS, P, and S produced 121,210 putative unigenes, of which only 59,199 unigenes (48.84%) had at least one significant match with an existing gene models in BLAST searches (Table 2). This result may be attributed to the absence of the completely sequenced genome for wheat.

Common wheat (2n = 6× = 42) has a large genome (16 Gb) and a high proportion of repetitive sequences (>80%); therefore, it is difficult to *de novo* assemble the hexaploid wheat transcriptome. Oono et al. [21] verified the three *de novo* assembly tools (e.g., Trans-Abyss, Velvet-Oases, and Trinity) by comparing analyses from several programs using short-read sequence data obtained from wheat cultivar CS seedlings grown under reduced phosphorus. Their results indicated that Trinity produced the largest number of contigs and the longest unigenes. In this study, 121,210 unigenes, which were *de novo* assembled by Trinity, were aligned against the draft sequence of the bread wheat genome (IWGSP 1.21), and 82.2% unigenes could be mapped to the exonic regions. This result further justified the use of Trinity to *de novo* assemble the hexaploid wheat transcriptome.

Our analysis identified 206 DEGs, each with differences greater than five-fold, which were common in the comparisons of PS *vs*. S and P *vs*. S. We deduced that these 206 genes may be highly correlated with stamen and pistil development because the PS and P have similar morphological structure and transcript profiles. The steady-state transcript levels for 25 genes were confirmed by qRT-PCR. Although the differences in gene expression based on qRT-PCR did not match the magnitude of those detected using RNA-seq, the upregulation and downregulation trends were similar. The lower expression levels detected by qRT-PCR could reflect the different sensitivities between the two technologies [25]. Illumina sequencing has been documented to be more sensitive for the estimation of gene expression, particularly for low-abundance transcripts, compared with microarrays and qRT-PCR [26]. Among the 206 genes, at least seven unigenes were previously reported to influence flower development: MIKC-type MADS-box transcription factor *WM27B* (*TaAG-4*), a *DL* related gene, *YAB1*, *YABBY 4*, *WM5*, *CER1*, and *WBLH1*.

The MADS-box transcription factor, *WM27*, belongs to the class D gene group and regulates ovule identity specification according to the “ABCDE” model in flower development [27]. In the present study, *WM27* expression was upregulated in PS and P. This result is similar to the finding of Paolacci et al. [28] that *TaAG-4* is highly expressed during late spike development, with weak expression in stamens, but high expression in pistils and caryopses. Therefore, the result demonstrated that WM27 may be involved in pistil development.

*YAB1*, the *DL* related gene, and *YABBY 4* belong to the YABBY gene family; their upregulated expressions were detected in PS and S. The genes of YABBY family in *Arabidopsis* determine the abaxial cell fate of lateral organs [29]. In rice, YAB1 plays a major role in meristem development and causes extra stamens and carpels [30]. Thus, YAB1 may have an important function in wheat pistil and stamen development. Yamaguchi et al. [31] indicated that the *DL* gene in rice interacts antagonistically with class B genes and controls floral meristem determinacy. Severe *DL* loss-of-function mutations caused the complete homeotic transformation of carpels into rice stamens. No literature has indicated that the *YABBY 4* gene is related to flower development. As a member of the YABBY gene family, YABBY 4 may have similar functions to other YABBY proteins. Moreover, the *YABBY 4* gene is upregulated in PS and P. Thus, YABBY 4 may contribute to pistil development.

Previous studies have shown that the wheat *Meiosis 5* (*WM5*) gene is expressed in young flowers during meiosis, and encodes a novel protein that appears to be involved in meristem development, and flower and pollen formation [32]. According to Dong et al. [32], expression of the *Meiosis 5* gene may be upregulated in stamens. However, *Meiosis 5* showed upregulated expression in PS and P. Thus, the function of Meiosis 5 in pistil and stamen development remains unclear and needs further experimental verification.

The *Arabidopsis CER1* gene encodes a novel protein involved in the conversion of long chain aldehydes to alkanes, which is a key step in wax biosynthesis. The *cer1* mutants, which are conditionally male sterile, display glossy green stems and fruit [33]. *CER1* was downregulated in PS and P or upregulated in S. This result indicated that CER1 is involved in stamen development. This result agrees with that of Aarts et al. [33].

The unigene comp122918_c0 is homologous to the wheat *BEL1* gene *WBLH1.* Plant *BEL1*-like homeobox (*BLH*) genes form a small gene family that functions in various developmental aspects, such as seed shattering in rice [34], or leaf shape establishment and ovule development in *Arabidopsis* [35,36]. Mizumoto et al. [37] isolated four *BLH* genes in wheat, designated *WBLH1* to *WBLH4*. Expression analysis indicated that the *WBLH1* gene is not related to ovule development. In the present study, the *WBLH1* gene was downregulated in PS and P (upregulated in S). This result agrees with the finding of Mizumoto et al., [37]. Therefore, we speculated that WBLH1 has an important function in stamen development. In addition to the seven unigenes, the other 199 genes probably also contribute to stamen and pistil development. However, further research is required to determine the mechanisms by which these genes control wheat stamen and pistil development.

## Conclusions

We sequenced and characterized the transcriptome of wheat PS, P from HTS-1 plants and S from the non-pistillody control CSTP. The use of this transcriptome resource enabled us to characterize gene expression profiles, examine differential expression profiles, and identify functional genes related to wheat stamen and pistil development. This work is significant for the development of genomic resources for wheat and provides important insights into the molecular mechanisms of wheat stamen and pistil development.

## Methods

### Plant materials and RNA extraction

HTS-1 is a novel common pistillody wheat mutant maintained in our laboratory. CSTP is a near-isogenic line of Chinese Spring, which is a common wheat variety that carries the *Pis1* gene derived from the TP mutant [38]. HTS-1 was selected during the development process of CSTP [6]. Thus, CSTP and HTS-1 are sib-lines that show similar phenotypes, except for pistillody. CSTP and HTS-1 were cultivated in a field in the China West Normal University, Nanchong, China. Ten HTS-1 plants and ten CSTP plants were pooled as two samples, respectively. The PS (Figure 1a) and P (Figure 1b) in the HTS-1 pool, and the S (Figure 1c) in the CSTP pool at the heading stage were selected for RNA extraction. The PS, P, and S were independently collected twice, creating two biological replicates.

RNA was isolated from PS, P, and S using a modified cetyl trimethylammonium bromide-based protocol [39], with high salt concentrations and was further purified with the RNeasy Plant Mini Kit (Qiagen, Shanghai, China). An equal amount of total RNA was pooled from each plant for each wheat line. RNA quality and quantity were determined using a NanoDrop ND-2000 Spectrophotometer (Nanodrop Technologies, Wilmington, DE, USA) and were verified for degradation using a 2100 Bioanalyser RNA Nanochip (Agilent, Palo Alto, CA, USA).

### Morphological observation

For the anatomical observations, the PS, P, and S in HTS-1 or CSTP at the heading stage were observed under a stereo microscope (Olympus, Tokyo, Japan). For light microscopy, the PS, P, and S were fixed in 50% ethanol, 0.9 mol/L glacial acetic acid and 3.7% formaldehyde at 4 °C for 15 h. The specimens were stained with Alcian blue and dehydrated through a graded ethanol series, infiltrated with xylene, and then embedded in paraffin. A 12-μm thick section was attached to the gelation-coated glass slides and observed under a light microscope (Olympus, Tokyo, Japan).

### cDNA library preparation and Illumina sequencing

Three micrograms of RNA per sample was used as the input material for the RNA sample preparations. Sequencing libraries were generated using NEB Next® Ultra™ Directional RNA Library Prep Kit for Illumina® (San Diego, CA, USA) following the recommendations of the manufacturer. Four index codes were added to assign sequences for each sample. In brief, mRNA was purified from total RNA using poly-T oligo-attached magnetic beads. Fragmentation was performed using divalent cations under elevated temperature in the Illumina proprietary fragmentation buffer. First-strand cDNA was synthesized using random oligonucleotides and SuperScript II. Second-strand cDNA synthesis was subsequently performed using DNA polymerase I and RNase H. The remaining overhangs were converted into blunt ends via exonuclease/polymerase activities and the enzymes were removed. After adenylation of the 3′ ends of DNA fragments, Illumina PE adapter oligonucleotides were ligated to prepare for hybridization. To select cDNA fragments of the preferred 200 bp in length, the library fragments were purified using an AMPure XP system (Beckman Coulter, Beverly, CA, USA). DNA fragments with ligated adaptor molecules on both ends were selectively enriched using Illumina PCR Primer Cocktail in a 12-cycle PCR reaction. The products were purified (AMPure XP system) and quantified using the Agilent high-sensitivity DNA assay on the Agilent Bioanalyzer 2100 system (Agilent, Santa Clara, CA, USA).

The clustering of the index-coded samples was performed on a cBot Cluster Generation System using TruSeq PE Cluster Kit v3-cBot-HS (Illumina), in accordance with the manufacturer’s instructions. After cluster generation, the library preparations were sequenced on an Illumina Hiseq 2000 platform and 100-bp paired-end reads were generated.

### *De novo* assembly

Raw reads of the fastq format were first processed through in-house Perl scripts. In this step, clean reads were obtained by removing reads that contained an adapter and vector contamination (the reads were screened against the Univec database http://www.ncbi.nlm.nih.gov/VecScreen/UniVec.html), reads that contained poly-N sequences (number of poly-Ns greater than 10%), and low-quality reads (Q20, Q30 and GC content were used for data filtering) from the raw data. The Trinity method [40] was used for the *de novo* assembly of wheat Illumina reads with the minimum kmer_cov set to 2 as the default, and all other parameters set to default. To avoid redundant transcripts, in-house Perl scripts were applied to extract the longest transcripts as unigenes. Unigenes generated with the assembly were used for downstream analysis. To evaluate the assembly strategies using Trinity, the unigenes were aligned to the draft sequence of the bread wheat genome (IWGSP 1.21, E value <10^−5^).

### Functional annotation

We annotated the unigenes based on a set of sequential BLAST searches, designed to find the most descriptive annotation for each sequence. The assembled unigenes were compared with sequences in the NCBI non-redundant (Nr) protein and nucleotide (Nt) databases (http://www.ncbi.nlm.nih.gov), the Protein family (Pram) database (http://en.wikipedia.org/wiki/Protein_family), the Cluster of Orthologous Group of proteins (KOG/COG) database (http://www.ncbi.nlm.nih.gov/COG), the Swiss-Prot protein database (http://www.expasy.ch/sprot), the KEGG Ortholog (KO) database (http://www.genome.jp/kegg/pathway.html), and the Gene Ontology (GO) database (http://www.geneontology.org/). Further functional enrichment analysis of DEGs was carried out using ToGO (Bioconductor package for R) (http://www.bioconductor.org/packages/release/bioc/html/topGO.html).

### Differential gene expression analysis

Before differential gene expression analysis, the read counts were adjusted using an edgeR program package for each sequenced library through one scaling normalized factor. A differential gene expression analysis of two samples (PS *vs*. P, PS *vs*. S, and P *vs*. S) was performed using the DEGseq R package. The p value was adjusted using a q value [41]. Q Value <0.005 and |log2 fold change| > 2 ware set as the threshold to judge the significance of gene expression difference. A large fold-change value (|log2 fold change| > 5) was also used to identify DEGs.

### Validation by qRT-PCR

Twenty-five primer pairs (Additional file 4: Table S3) were designed to generate amplicons to validate the RNA-seq data. Aliquots of the total RNA extracted for sequencing were used for quantitative real-time PCR experiments in accordance with manufacturer’s instructions (Qiagen, Shanghai, China). Real-time assays were performed with SYBR Green Dye (Takara, Dalian, China) using a Bio-Rad CFX96 real-time PCR platform (Bio-Rad Laboratories, Hercules, CA, USA). All assays for a particular gene were performed three times synchronously under identical conditions, and RNA transcript fold changes were calculated through the 2^-ΔΔCt^method [42] with the wheat housekeeping genes Ubiq (DQ086482) and Actin (AB181911) as internal controls [13,43].

## Additional files

Additional file 1: Figure S1. Distribution of assembly transcripts and unigenes lengths. Additional file 2: Table S1. KEGG pathways represented in PS, P and S. Additional file 3: Table S2. The common differentially expressed genes between PS vs S and P vs S. Additional file 4: Table S3. The primers designed for qRT-PCR.
