# Supplementary figures and images for: Pistillody mutant reveals key insights into stamen and pistil development in wheat (Triticum aestivum L.)
Source: BMC Genomics. 2015 Mar 19;16(1):211. doi: 10.1186/s12864-015-1453-0 (PMC4369888; doi:10.1186/s12864-015-1453-0)

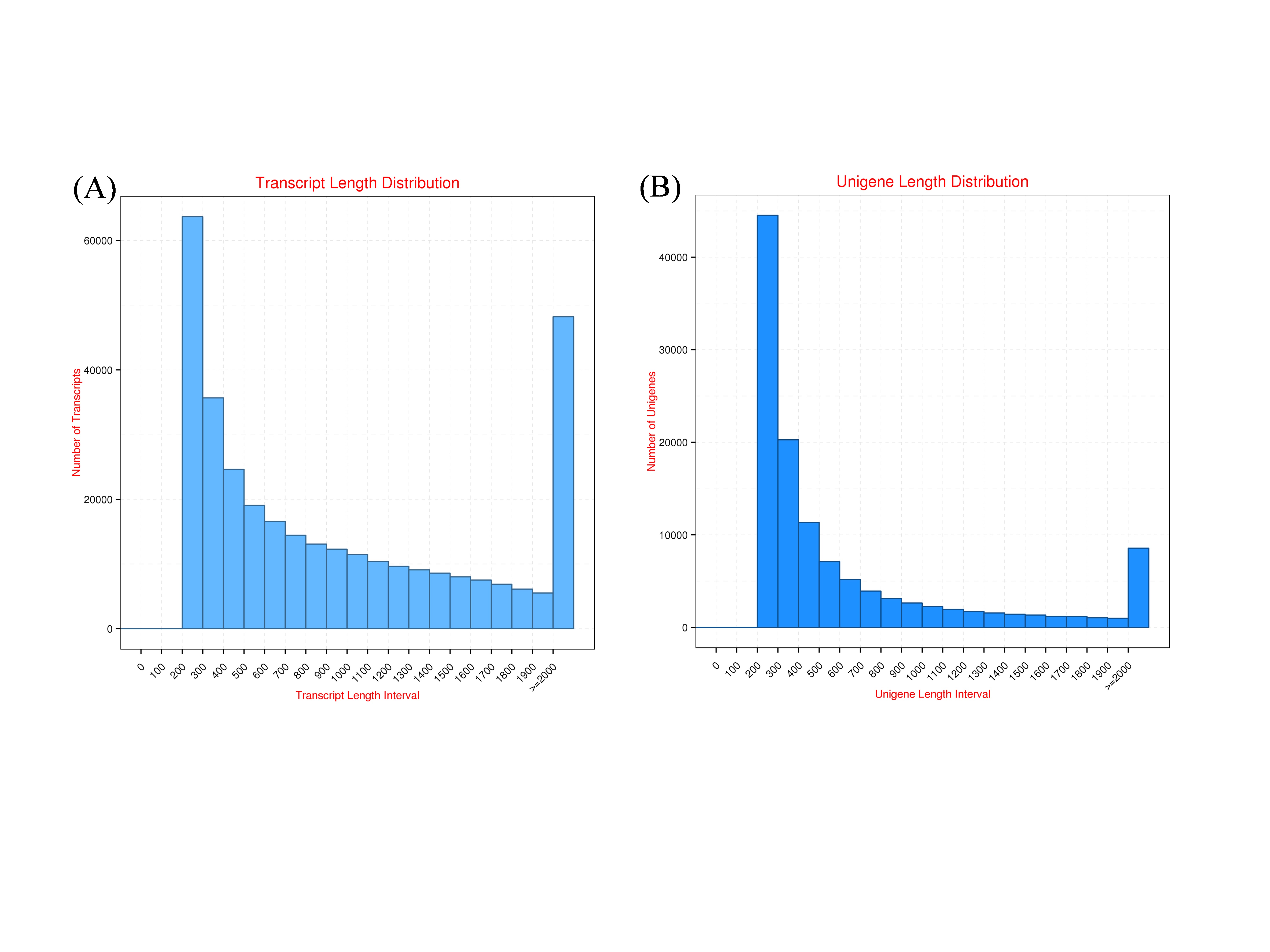

Supplement: Additional file 1: Figure S1. — Distribution of assembly transcripts and unigenes lengths. [file 12864_2015_1453_MOESM1_ESM.jpeg]
