# Supplementary material for: Pistillody mutant reveals key insights into stamen and pistil development in wheat (Triticum aestivum L.)
Source: BMC Genomics. 2015 Mar 19;16(1):211. doi: 10.1186/s12864-015-1453-0 (PMC4369888; doi:10.1186/s12864-015-1453-0)
Supplement: Additional file 2: Table S1. — KEGG pathways represented in PS, P and S. [file 12864_2015_1453_MOESM2_ESM.docx]

Table S1 KEGG pathways represented in PS, P and S

| Pathway Hierarchy 1 | Pathway Hierarchy 2 | KEGG Pathway | Pathway ID | Gene Number |
| --- | --- | --- | --- | --- |
| Cellular Processes | Cell communication | Adherens junction | ko04520 | 46 |
| Cellular Processes | Cell communication | Focal adhesion | ko04510 | 48 |
| Cellular Processes | Cell communication | Gap junction | ko04540 | 24 |
| Cellular Processes | Cell communication | Tight junction | ko04530 | 35 |
| Cellular Processes | Cell growth and death | Apoptosis | ko04210 | 10 |
| Cellular Processes | Cell growth and death | Cell cycle | ko04110 | 149 |
| Cellular Processes | Cell growth and death | Cell cycle - Caulobacter | ko04112 | 15 |
| Cellular Processes | Cell growth and death | Cell cycle - yeast | ko04111 | 99 |
| Cellular Processes | Cell growth and death | Meiosis - yeast | ko04113 | 73 |
| Cellular Processes | Cell growth and death | Oocyte meiosis | ko04114 | 104 |
| Cellular Processes | Cell growth and death | p53 signaling pathway | ko04115 | 37 |
| Cellular Processes | Cell motility | Regulation of actin cytoskeleton | ko04810 | 98 |
| Cellular Processes | Transport and catabolism | Endocytosis | ko04144 | 158 |
| Cellular Processes | Transport and catabolism | Lysosome | ko04142 | 73 |
| Cellular Processes | Transport and catabolism | Peroxisome | ko04146 | 99 |
| Cellular Processes | Transport and catabolism | Phagosome | ko04145 | 119 |
| Cellular Processes | Transport and catabolism | Regulation of autophagy | ko04140 | 25 |
| Environmental Information Processing | Membrane transport | ABC transporters | ko02010 | 53 |
| Environmental Information Processing | Membrane transport | Bacterial secretion system | ko03070 | 23 |
| Environmental Information Processing | Signal transduction | Calcium signaling pathway | ko04020 | 42 |
| Environmental Information Processing | Signal transduction | ErbB signaling pathway | ko04012 | 28 |
| Environmental Information Processing | Signal transduction | HIF-1 signaling pathway | ko04066 | 76 |
| Environmental Information Processing | Signal transduction | Hedgehog signaling pathway | ko04340 | 22 |
| Environmental Information Processing | Signal transduction | Hippo signaling pathway | ko04390 | 53 |
| Environmental Information Processing | Signal transduction | Hippo signaling pathway -fly | ko04391 | 33 |
| Environmental Information Processing | Signal transduction | Jak-STAT signaling pathway | ko04630 | 13 |
| Environmental Information Processing | Signal transduction | MAPK signaling pathway | ko04010 | 51 |
| Environmental Information Processing | Signal transduction | MAPK signaling pathway - fly | ko04013 | 8 |
| Environmental Information Processing | Signal transduction | MAPK signaling pathway - yeast | ko04011 | 11 |
| Environmental Information Processing | Signal transduction | NF-kappa B signaling pathway | ko04064 | 17 |
| Environmental Information Processing | Signal transduction | Notch signaling pathway | ko04330 | 23 |
| Environmental Information Processing | Signal transduction | PI3K-Akt signaling pathway | ko04151 | 94 |
| Environmental Information Processing | Signal transduction | Phosphatidylinositol signaling system | ko04070 | 75 |
| Environmental Information Processing | Signal transduction | Plant hormone signal transduction | ko04075 | 202 |
| Environmental Information Processing | Signal transduction | TGF-beta signaling pathway | ko04350 | 49 |
| Environmental Information Processing | Signal transduction | Two-component system | ko02020 | 23 |
| Environmental Information Processing | Signal transduction | VEGF signaling pathway | ko04370 | 23 |
| Environmental Information Processing | Signal transduction | Wnt signaling pathway | ko04310 | 83 |
| Environmental Information Processing | Signal transduction | mTOR signaling pathway | ko04150 | 33 |
| Environmental Information Processing | Signaling molecules and interaction | Neuroactive ligand-receptor interaction | ko04080 | 9 |
| Genetic Information Processing | Folding, sorting and degradation | Proteasome | ko03050 | 68 |
| Genetic Information Processing | Folding, sorting and degradation | Protein export | ko03060 | 79 |
| Genetic Information Processing | Folding, sorting and degradation | Protein processing in endoplasmic reticulum | ko04141 | 298 |
| Genetic Information Processing | Folding, sorting and degradation | RNA degradation | ko03018 | 127 |
| Genetic Information Processing | Folding, sorting and degradation | SNARE interactions in vesicular transport | ko04130 | 40 |
| Genetic Information Processing | Folding, sorting and degradation | Sulfur relay system | ko04122 | 14 |
| Genetic Information Processing | Folding, sorting and degradation | Ubiquitin mediated proteolysis | ko04120 | 179 |
| Genetic Information Processing | Replication and repair | Base excision repair | ko03410 | 43 |
| Genetic Information Processing | Replication and repair | DNA replication | ko03030 | 61 |
| Genetic Information Processing | Replication and repair | Fanconi anemia pathway | ko03460 | 71 |
| Genetic Information Processing | Replication and repair | Homologous recombination | ko03440 | 67 |
| Genetic Information Processing | Replication and repair | Mismatch repair | ko03430 | 50 |
| Genetic Information Processing | Replication and repair | Non-homologous end-joining | ko03450 | 8 |
| Genetic Information Processing | Replication and repair | Nucleotide excision repair | ko03420 | 75 |
| Genetic Information Processing | Transcription | Basal transcription factors | ko03022 | 47 |
| Genetic Information Processing | Transcription | RNA polymerase | ko03020 | 61 |
| Genetic Information Processing | Transcription | Spliceosome | ko03040 | 297 |
| Genetic Information Processing | Translation | Aminoacyl-tRNA biosynthesis | ko00970 | 125 |
| Genetic Information Processing | Translation | RNA transport | ko03013 | 245 |
| Genetic Information Processing | Translation | Ribosome | ko03010 | 324 |
| Genetic Information Processing | Translation | Ribosome biogenesis in eukaryotes | ko03008 | 111 |
| Genetic Information Processing | Translation | mRNA surveillance pathway | ko03015 | 129 |
| Metabolism | Amino acid metabolism | Alanine, aspartate and glutamate metabolism | ko00250 | 57 |
| Metabolism | Amino acid metabolism | Arginine and proline metabolism | ko00330 | 89 |
| Metabolism | Amino acid metabolism | Cysteine and methionine metabolism | ko00270 | 109 |
| Metabolism | Amino acid metabolism | Glycine, serine and threonine metabolism | ko00260 | 66 |
| Metabolism | Amino acid metabolism | Histidine metabolism | ko00340 | 23 |
| Metabolism | Amino acid metabolism | Lysine biosynthesis | ko00300 | 22 |
| Metabolism | Amino acid metabolism | Lysine degradation | ko00310 | 46 |
| Metabolism | Amino acid metabolism | Phenylalanine metabolism | ko00360 | 125 |
| Metabolism | Amino acid metabolism | Phenylalanine, tyrosine and tryptophan biosynthesis | ko00400 | 49 |
| Metabolism | Amino acid metabolism | Tryptophan metabolism | ko00380 | 55 |
| Metabolism | Amino acid metabolism | Tyrosine metabolism | ko00350 | 53 |
| Metabolism | Amino acid metabolism | Valine, leucine and isoleucine biosynthesis | ko00290 | 16 |
| Metabolism | Amino acid metabolism | Valine, leucine and isoleucine degradation | ko00280 | 44 |
| Metabolism | Biosynthesis of other secondary metabolites | Betalain biosynthesis | ko00965 | 5 |
| Metabolism | Biosynthesis of other secondary metabolites | Butirosin and neomycin biosynthesis | ko00524 | 9 |
| Metabolism | Biosynthesis of other secondary metabolites | Caffeine metabolism | ko00232 | 6 |
| Metabolism | Biosynthesis of other secondary metabolites | Flavone and flavonol biosynthesis | ko00944 | 17 |
| Metabolism | Biosynthesis of other secondary metabolites | Flavonoid biosynthesis | ko00941 | 55 |
| Metabolism | Biosynthesis of other secondary metabolites | Glucosinolate biosynthesis | ko00966 | 1 |
| Metabolism | Biosynthesis of other secondary metabolites | Isoquinoline alkaloid biosynthesis | ko00950 | 25 |
| Metabolism | Biosynthesis of other secondary metabolites | Novobiocin biosynthesis | ko00401 | 6 |
| Metabolism | Biosynthesis of other secondary metabolites | Phenylpropanoid biosynthesis | ko00940 | 172 |
| Metabolism | Biosynthesis of other secondary metabolites | Stilbenoid, diarylheptanoid and gingerol biosynthesis | ko00945 | 30 |
| Metabolism | Biosynthesis of other secondary metabolites | Streptomycin biosynthesis | ko00521 | 17 |
| Metabolism | Biosynthesis of other secondary metabolites | Tropane, piperidine and pyridine alkaloid biosynthesis | ko00960 | 22 |
| Metabolism | Carbohydrate metabolism | Amino sugar and nucleotide sugar metabolism | ko00520 | 136 |
| Metabolism | Carbohydrate metabolism | Ascorbate and aldarate metabolism | ko00053 | 57 |
| Metabolism | Carbohydrate metabolism | Butanoate metabolism | ko00650 | 30 |
| Metabolism | Carbohydrate metabolism | C5-Branched dibasic acid metabolism | ko00660 | 6 |
| Metabolism | Carbohydrate metabolism | Citrate cycle (TCA cycle) | ko00020 | 62 |
| Metabolism | Carbohydrate metabolism | Fructose and mannose metabolism | ko00051 | 87 |
| Metabolism | Carbohydrate metabolism | Galactose metabolism | ko00052 | 108 |
| Metabolism | Carbohydrate metabolism | Glycolysis / Gluconeogenesis | ko00010 | 190 |
| Metabolism | Carbohydrate metabolism | Glyoxylate and dicarboxylate metabolism | ko00630 | 86 |
| Metabolism | Carbohydrate metabolism | Inositol phosphate metabolism | ko00562 | 74 |
| Metabolism | Carbohydrate metabolism | Pentose and glucuronate interconversions | ko00040 | 71 |
| Metabolism | Carbohydrate metabolism | Pentose phosphate pathway | ko00030 | 79 |
| Metabolism | Carbohydrate metabolism | Propanoate metabolism | ko00640 | 42 |
| Metabolism | Carbohydrate metabolism | Pyruvate metabolism | ko00620 | 107 |
| Metabolism | Carbohydrate metabolism | Starch and sucrose metabolism | ko00500 | 260 |
| Metabolism | Energy metabolism | Carbon fixation in photosynthetic organisms | ko00710 | 117 |
| Metabolism | Energy metabolism | Carbon fixation pathways in prokaryotes | ko00720 | 40 |
| Metabolism | Energy metabolism | Methane metabolism | ko00680 | 168 |
| Metabolism | Energy metabolism | Nitrogen metabolism | ko00910 | 61 |
| Metabolism | Energy metabolism | Oxidative phosphorylation | ko00190 | 192 |
| Metabolism | Energy metabolism | Photosynthesis | ko00195 | 71 |
| Metabolism | Energy metabolism | Photosynthesis - antenna proteins | ko00196 | 37 |
| Metabolism | Energy metabolism | Sulfur metabolism | ko00920 | 31 |
| Metabolism | Glycan biosynthesis and metabolism | Glycosaminoglycan degradation | ko00531 | 10 |
| Metabolism | Glycan biosynthesis and metabolism | Glycosphingolipid biosynthesis - ganglio series | ko00604 | 6 |
| Metabolism | Glycan biosynthesis and metabolism | Glycosphingolipid biosynthesis - globo series | ko00603 | 9 |
| Metabolism | Glycan biosynthesis and metabolism | Glycosylphosphatidylinositol(GPI)-anchor biosynthesis | ko00563 | 22 |
| Metabolism | Glycan biosynthesis and metabolism | Lipopolysaccharide biosynthesis | ko00540 | 5 |
| Metabolism | Glycan biosynthesis and metabolism | N-Glycan biosynthesis | ko00510 | 38 |
| Metabolism | Glycan biosynthesis and metabolism | Other glycan degradation | ko00511 | 16 |
| Metabolism | Glycan biosynthesis and metabolism | Other types of O-glycan biosynthesis | ko00514 | 2 |
| Metabolism | Glycan biosynthesis and metabolism | Peptidoglycan biosynthesis | ko00550 | 1 |
| Metabolism | Glycan biosynthesis and metabolism | Various types of N-glycan biosynthesis | ko00513 | 30 |
| Metabolism | Lipid metabolism | Arachidonic acid metabolism | ko00590 | 11 |
| Metabolism | Lipid metabolism | Biosynthesis of unsaturated fatty acids | ko01040 | 43 |
| Metabolism | Lipid metabolism | Cutin, suberine and wax biosynthesis | ko00073 | 31 |
| Metabolism | Lipid metabolism | Ether lipid metabolism | ko00565 | 43 |
| Metabolism | Lipid metabolism | Fatty acid biosynthesis | ko00061 | 41 |
| Metabolism | Lipid metabolism | Fatty acid elongation | ko00062 | 37 |
| Metabolism | Lipid metabolism | Fatty acid metabolism | ko00071 | 69 |
| Metabolism | Lipid metabolism | Glycerolipid metabolism | ko00561 | 74 |
| Metabolism | Lipid metabolism | Glycerophospholipid metabolism | ko00564 | 105 |
| Metabolism | Lipid metabolism | Linoleic acid metabolism | ko00591 | 37 |
| Metabolism | Lipid metabolism | Sphingolipid metabolism | ko00600 | 21 |
| Metabolism | Lipid metabolism | Steroid biosynthesis | ko00100 | 27 |
| Metabolism | Lipid metabolism | Steroid hormone biosynthesis | ko00140 | 5 |
| Metabolism | Lipid metabolism | Synthesis and degradation of ketone bodies | ko00072 | 7 |
| Metabolism | Lipid metabolism | alpha-Linolenic acid metabolism | ko00592 | 53 |
| Metabolism | Metabolism of cofactors and vitamins | Biotin metabolism | ko00780 | 17 |
| Metabolism | Metabolism of cofactors and vitamins | Folate biosynthesis | ko00790 | 11 |
| Metabolism | Metabolism of cofactors and vitamins | Lipoic acid metabolism | ko00785 | 4 |
| Metabolism | Metabolism of cofactors and vitamins | Nicotinate and nicotinamide metabolism | ko00760 | 15 |
| Metabolism | Metabolism of cofactors and vitamins | One carbon pool by folate | ko00670 | 22 |
| Metabolism | Metabolism of cofactors and vitamins | Pantothenate and CoA biosynthesis | ko00770 | 27 |
| Metabolism | Metabolism of cofactors and vitamins | Porphyrin and chlorophyll metabolism | ko00860 | 61 |
| Metabolism | Metabolism of cofactors and vitamins | Retinol metabolism | ko00830 | 22 |
| Metabolism | Metabolism of cofactors and vitamins | Riboflavin metabolism | ko00740 | 8 |
| Metabolism | Metabolism of cofactors and vitamins | Thiamine metabolism | ko00730 | 14 |
| Metabolism | Metabolism of cofactors and vitamins | Ubiquinone and other terpenoid-quinone biosynthesis | ko00130 | 49 |
| Metabolism | Metabolism of cofactors and vitamins | Vitamin B6 metabolism | ko00750 | 14 |
| Metabolism | Metabolism of other amino acids | Cyanoamino acid metabolism | ko00460 | 47 |
| Metabolism | Metabolism of other amino acids | D-Glutamine and D-glutamate metabolism | ko00471 | 4 |
| Metabolism | Metabolism of other amino acids | Glutathione metabolism | ko00480 | 105 |
| Metabolism | Metabolism of other amino acids | Phosphonate and phosphinate metabolism | ko00440 | 8 |
| Metabolism | Metabolism of other amino acids | Selenocompound metabolism | ko00450 | 35 |
| Metabolism | Metabolism of other amino acids | Taurine and hypotaurine metabolism | ko00430 | 13 |
| Metabolism | Metabolism of other amino acids | beta-Alanine metabolism | ko00410 | 37 |
| Metabolism | Metabolism of terpenoids and polyketides | Biosynthesis of ansamycins | ko01051 | 7 |
| Metabolism | Metabolism of terpenoids and polyketides | Biosynthesis of siderophore group nonribosomal peptides | ko01053 | 2 |
| Metabolism | Metabolism of terpenoids and polyketides | Brassinosteroid biosynthesis | ko00905 | 13 |
| Metabolism | Metabolism of terpenoids and polyketides | Carotenoid biosynthesis | ko00906 | 27 |
| Metabolism | Metabolism of terpenoids and polyketides | Diterpenoid biosynthesis | ko00904 | 19 |
| Metabolism | Metabolism of terpenoids and polyketides | Geraniol degradation | ko00281 | 1 |
| Metabolism | Metabolism of terpenoids and polyketides | Limonene and pinene degradation | ko00903 | 22 |
| Metabolism | Metabolism of terpenoids and polyketides | Monoterpenoid biosynthesis | ko00902 | 4 |
| Metabolism | Metabolism of terpenoids and polyketides | Polyketide sugar unit biosynthesis | ko00523 | 1 |
| Metabolism | Metabolism of terpenoids and polyketides | Sesquiterpenoid and triterpenoid biosynthesis | ko00909 | 3 |
| Metabolism | Metabolism of terpenoids and polyketides | Terpenoid backbone biosynthesis | ko00900 | 59 |
| Metabolism | Metabolism of terpenoids and polyketides | Tetracycline biosynthesis | ko00253 | 2 |
| Metabolism | Metabolism of terpenoids and polyketides | Zeatin biosynthesis | ko00908 | 21 |
| Metabolism | Nucleotide metabolism | Purine metabolism | ko00230 | 177 |
| Metabolism | Nucleotide metabolism | Pyrimidine metabolism | ko00240 | 142 |
| Metabolism | Xenobiotics biodegradation and metabolism | 1,1,1-Trichloro-2,2-bis(4-chlorophenyl)ethane (DDT) degradation | ko00351 | 2 |
| Metabolism | Xenobiotics biodegradation and metabolism | Aminobenzoate degradation | ko00627 | 9 |
| Metabolism | Xenobiotics biodegradation and metabolism | Benzoate degradation | ko00362 | 9 |
| Metabolism | Xenobiotics biodegradation and metabolism | Bisphenol degradation | ko00363 | 9 |
| Metabolism | Xenobiotics biodegradation and metabolism | Caprolactam degradation | ko00930 | 2 |
| Metabolism | Xenobiotics biodegradation and metabolism | Chloroalkane and chloroalkene degradation | ko00625 | 33 |
| Metabolism | Xenobiotics biodegradation and metabolism | Chlorocyclohexane and chlorobenzene degradation | ko00361 | 7 |
| Metabolism | Xenobiotics biodegradation and metabolism | Drug metabolism - cytochrome P450 | ko00982 | 61 |
| Metabolism | Xenobiotics biodegradation and metabolism | Drug metabolism - other enzymes | ko00983 | 21 |
| Metabolism | Xenobiotics biodegradation and metabolism | Fluorobenzoate degradation | ko00364 | 5 |
| Metabolism | Xenobiotics biodegradation and metabolism | Metabolism of xenobiotics by cytochrome P450 | ko00980 | 61 |
| Metabolism | Xenobiotics biodegradation and metabolism | Naphthalene degradation | ko00626 | 19 |
| Metabolism | Xenobiotics biodegradation and metabolism | Polycyclic aromatic hydrocarbon degradation | ko00624 | 10 |
| Metabolism | Xenobiotics biodegradation and metabolism | Styrene degradation | ko00643 | 5 |
| Metabolism | Xenobiotics biodegradation and metabolism | Toluene degradation | ko00623 | 5 |
| Organismal Systems | Circulatory system | Cardiac muscle contraction | ko04260 | 32 |
| Organismal Systems | Circulatory system | Vascular smooth muscle contraction | ko04270 | 30 |
| Organismal Systems | Development | Axon guidance | ko04360 | 40 |
| Organismal Systems | Development | Dorso-ventral axis formation | ko04320 | 8 |
| Organismal Systems | Development | Osteoclast differentiation | ko04380 | 21 |
| Organismal Systems | Digestive system | Bile secretion | ko04976 | 53 |
| Organismal Systems | Digestive system | Carbohydrate digestion and absorption | ko04973 | 17 |
| Organismal Systems | Digestive system | Fat digestion and absorption | ko04975 | 7 |
| Organismal Systems | Digestive system | Gastric acid secretion | ko04971 | 13 |
| Organismal Systems | Digestive system | Mineral absorption | ko04978 | 15 |
| Organismal Systems | Digestive system | Pancreatic secretion | ko04972 | 15 |
| Organismal Systems | Digestive system | Protein digestion and absorption | ko04974 | 10 |
| Organismal Systems | Digestive system | Salivary secretion | ko04970 | 13 |
| Organismal Systems | Digestive system | Vitamin digestion and absorption | ko04977 | 1 |
| Organismal Systems | Endocrine system | Adipocytokine signaling pathway | ko04920 | 25 |
| Organismal Systems | Endocrine system | GnRH signaling pathway | ko04912 | 52 |
| Organismal Systems | Endocrine system | Insulin secretion | ko04911 | 1 |
| Organismal Systems | Endocrine system | Insulin signaling pathway | ko04910 | 114 |
| Organismal Systems | Endocrine system | Melanogenesis | ko04916 | 44 |
| Organismal Systems | Endocrine system | PPAR signaling pathway | ko03320 | 56 |
| Organismal Systems | Endocrine system | Progesterone-mediated oocyte maturation | ko04914 | 49 |
| Organismal Systems | Endocrine system | Renin-angiotensin system | ko04614 | 5 |
| Organismal Systems | Environmental adaptation | Circadian entrainment | ko04713 | 21 |
| Organismal Systems | Environmental adaptation | Circadian rhythm | ko04710 | 28 |
| Organismal Systems | Environmental adaptation | Circadian rhythm - fly | ko04711 | 14 |
| Organismal Systems | Environmental adaptation | Circadian rhythm - plant | ko04712 | 41 |
| Organismal Systems | Environmental adaptation | Plant-pathogen interaction | ko04626 | 226 |
| Organismal Systems | Excretory system | Aldosterone-regulated sodium reabsorption | ko04960 | 8 |
| Organismal Systems | Excretory system | Collecting duct acid secretion | ko04966 | 32 |
| Organismal Systems | Excretory system | Endocrine and other factor-regulated calcium reabsorption | ko04961 | 19 |
| Organismal Systems | Excretory system | Proximal tubule bicarbonate reclamation | ko04964 | 5 |
| Organismal Systems | Excretory system | Vasopressin-regulated water reabsorption | ko04962 | 12 |
| Organismal Systems | Immune system | Antigen processing and presentation | ko04612 | 81 |
| Organismal Systems | Immune system | B cell receptor signaling pathway | ko04662 | 32 |
| Organismal Systems | Immune system | Chemokine signaling pathway | ko04062 | 27 |
| Organismal Systems | Immune system | Cytosolic DNA-sensing pathway | ko04623 | 34 |
| Organismal Systems | Immune system | Fc epsilon RI signaling pathway | ko04664 | 15 |
| Organismal Systems | Immune system | Fc gamma R-mediated phagocytosis | ko04666 | 99 |
| Organismal Systems | Immune system | Hematopoietic cell lineage | ko04640 | 3 |
| Organismal Systems | Immune system | Leukocyte transendothelial migration | ko04670 | 19 |
| Organismal Systems | Immune system | NOD-like receptor signaling pathway | ko04621 | 24 |
| Organismal Systems | Immune system | Natural killer cell mediated cytotoxicity | ko04650 | 21 |
| Organismal Systems | Immune system | RIG-I-like receptor signaling pathway | ko04622 | 11 |
| Organismal Systems | Immune system | T cell receptor signaling pathway | ko04660 | 25 |
| Organismal Systems | Immune system | Toll-like receptor signaling pathway | ko04620 | 18 |
| Organismal Systems | Nervous system | Cholinergic synapse | ko04725 | 9 |
| Organismal Systems | Nervous system | Dopaminergic synapse | ko04728 | 52 |
| Organismal Systems | Nervous system | GABAergic synapse | ko04727 | 27 |
| Organismal Systems | Nervous system | Glutamatergic synapse | ko04724 | 54 |
| Organismal Systems | Nervous system | Long-term depression | ko04730 | 13 |
| Organismal Systems | Nervous system | Long-term potentiation | ko04720 | 47 |
| Organismal Systems | Nervous system | Neurotrophin signaling pathway | ko04722 | 51 |
| Organismal Systems | Nervous system | Retrograde endocannabinoid signaling | ko04723 | 13 |
| Organismal Systems | Nervous system | Serotonergic synapse | ko04726 | 9 |
| Organismal Systems | Nervous system | Synaptic vesicle cycle | ko04721 | 65 |
| Organismal Systems | Sensory system | Olfactory transduction | ko04740 | 13 |
| Organismal Systems | Sensory system | Phototransduction | ko04744 | 14 |
| Organismal Systems | Sensory system | Phototransduction - fly | ko04745 | 24 |
| Organismal Systems | Sensory system | Taste transduction | ko04742 | 1 |
