# Supplementary material for: Pistillody mutant reveals key insights into stamen and pistil development in wheat (Triticum aestivum L.)
Source: BMC Genomics. 2015 Mar 19;16(1):211. doi: 10.1186/s12864-015-1453-0 (PMC4369888; doi:10.1186/s12864-015-1453-0)
Supplement: Additional file 3: Table S2. — The common differentially expressed genes between PS vs S and P vs S. [file 12864_2015_1453_MOESM3_ESM.docx]

Table S2 The common differentially expressed genes between PS vs S and P vs S

| Gene | log2 ratio  (PS VS S) | log2 ratio  (P VS S) | Description |
| --- | --- | --- | --- |
| UP-regulated genes |  |  |  |
| comp103079_c0 | 6.40 | 6.91 | bphyem107m16, full insert sequence [*Phyllostachys edulis*] |
| comp103277_c0 | 8.97 | 6.72 | *Triticum aestivum* cDNA, clone: WT004_L02, cultivar: Chinese Spring [*Triticum aestivum*] |
| comp103862_c0 | 8.36 | 8.41 |  |
| comp104128_c0 | 7.64 | 7.36 |  |
| comp105479_c0 | 6.88 | 7.18 | predicted protein [*Hordeum vulgare*] |
| comp107249_c0 | 7.32 | 6.78 | Os08g0220400 [*Oryza sativa*] |
| comp107730_c0 | 5.79 | 6.91 |  |
| comp107730_c1 | 8.12 | 8.90 |  |
| comp108229_c0 | 6.10 | 6.83 | MIKC-type MADS-box transcription factor WM27B [*Triticum aestivum*] |
| comp108539_c0 | 5.83 | 5.28 | predicted protein [*Hordeum vulgare*] |
| comp108901_c0 | 8.60 | 9.19 |  |
| comp109228_c0 | 8.93 | 8.98 | predicted protein [*Hordeum vulgare*] |
| comp109492_c0 | 6.87 | 7.25 | EPIDERMAL PATTERNING FACTOR-like protein 1-like [*Brachypodium distachyon*] |
| comp110066_c0 | 7.96 | 8.50 | DL related protein [*Triticum aestivum*] |
| comp110106_c0 | 5.99 | 7.52 | YABBY protein [*Triticum aestivum*] |
| comp110264_c0 | 7.02 | 7.24 | predicted protein [*Hordeum vulgare*] |
| comp110264_c1 | 5.75 | 6.39 | predicted protein [*Hordeum vulgare*] |
| comp110651_c0 | 5.42 | 6.16 | predicted protein [*Hordeum vulgare*] |
| comp110936_c0 | 7.96 | 6.24 | predicted protein [*Hordeum vulgare*] |
| comp111376_c0 | 5.08 | 5.62 | hypothetical protein OsJ_08458 [*Oryza sativa*] |
| comp111868_c0 | 6.38 | 6.45 | Os10g0544600 [*Oryza sativa*] |
| comp111949_c1 | 6.80 | 7.17 | predicted protein [*Hordeum vulgare*] |
| comp112313_c0 | 5.40 | 5.97 | protein YABBY 4-like [*Brachypodium distachyon*] |
| comp113007_c0 | 5.23 | 5.02 | meiosis 5 [*Triticum aestivum*] |
| comp113041_c0 | 8.11 | 8.17 | Os03g0212300 [*Oryza sativa*] |
| comp113043_c0 | 7.52 | 8.29 | predicted protein [*Hordeum vulgare*] |
| comp113043_c1 | 5.02 | 5.03 | OsPIN3a gene for auxin efflux carrier [*Oryza sativa*] |
| comp113050_c0 | 7.51 | 8.00 | probable polygalacturonase At1g80170-like [*Brachypodium distachyon*] |
| comp113062_c0 | 7.43 | 8.80 | predicted protein [*Hordeum vulgare*] |
| comp113292_c0 | 5.65 | 7.21 | purple acid phosphatase 4-like [*Brachypodium distachyon*] |
| comp113310_c0 | 6.15 | 5.46 | predicted protein [*Hordeum vulgare*] |
| comp113918_c0 | 5.14 | 5.80 | predicted protein [*Hordeum vulgare*] |
| comp114327_c0 | 5.32 | 5.83 | hypothetical protein ZEAMMB73_856541 [*Zea mays*] |
| comp115245_c0 | 8.17 | 6.07 | endochitinase [*Triticum aestivum*] |
| comp115385_c0 | 5.37 | 5.52 | fasciclin-like protein FLA33 [*Triticum aestivum*] |
| comp116703_c0 | 6.16 | 6.31 | predicted protein [*Hordeum vulgare*] |
| comp116835_c0 | 9.70 | 8.84 | cytokinin oxidase/dehydrogenase [*Triticum aestivum*] |
| comp117062_c0 | 6.80 | 7.32 | probable protein phosphatase 2C 8-like [*Brachypodium distachyon*] |
| comp117360_c0 | 5.52 | 5.66 | predicted protein [*Hordeum vulgare*] |
| comp117613_c1 | 6.23 | 5.63 | ADP-glucose pyrophosphorylase [*Triticum aestivum*] |
| comp117947_c0 | 5.04 | 5.02 | 2-aminoethanethiol dioxygenase-like [*Brachypodium distachyon*] |
| comp118341_c0 | 5.46 | 6.30 | predicted protein [*Hordeum vulgare*] |
| comp118851_c1 | 5.05 | 5.14 | BTB/POZ domain-containing protein At3g08570-like [*Brachypodium distachyon*] |
| comp119100_c0 | 5.68 | 5.84 | predicted protein [*Hordeum vulgare*] |
| comp119135_c0 | 5.38 | 6.55 | predicted protein [*Hordeum vulgare*] |
| comp119301_c0 | 5.34 | 5.28 | predicted protein [*Hordeum vulgare*] |
| comp119404_c0 | 5.47 | 6.16 | inositol-3-phosphate synthase-like [*Brachypodium distachyon*] |
| comp119576_c0 | 5.86 | 6.33 | two-component system protein A-like [*Brachypodium distachyon*] |
| comp120749_c0 | 5.03 | 5.74 | uncharacterized protein LOC100845608 [*Brachypodium distachyon*] |
| comp121034_c0 | 5.30 | 5.69 | predicted protein [*Hordeum vulgare*] |
| comp121204_c0 | 8.09 | 7.60 | predicted protein [*Hordeum vulgare*] |
| comp122072_c0 | 6.56 | 6.55 | predicted protein [*Hordeum vulgare*] |
| comp122074_c0 | 5.62 | 5.95 | uncharacterized protein LOC100842951 [*Brachypodium distachyon*] |
| comp122566_c0 | 5.30 | 5.33 | uncharacterized GPI-anchored protein At4g28100-like [*Brachypodium distachyon*] |
| comp123058_c0 | 5.29 | 5.24 | uncharacterized protein LOC100823571 [*Brachypodium distachyon*] |
| comp123359_c0 | 5.83 | 5.94 | predicted protein [*Hordeum vulgare*] |
| comp123386_c1 | 5.64 | 5.27 | predicted protein [*Hordeum vulgare]* |
| comp123488_c0 | 5.38 | 5.01 | predicted protein [*Hordeum vulgare*] |
| comp125818_c0 | 8.80 | 8.11 |  |
| comp126922_c0 | 8.57 | 8.45 |  |
| comp128980_c0 | 6.10 | 5.64 |  |
| comp138318_c0 | 5.04 | 6.16 |  |
| comp144486_c0 | 5.15 | 5.32 | predicted protein [*Hordeum vulgare*] |
| comp146808_c0 | 5.13 | 5.62 |  |
| comp36800_c0 | 6.27 | 6.66 | predicted protein [*Hordeum vulgare*] |
| comp41682_c0 | 8.89 | 9.17 |  |
| comp62166_c0 | 7.45 | 7.48 | Os01g0597600 [*Oryza sativa*] |
| comp79245_c0 | 9.00 | 8.72 |  |
| comp83842_c0 | 5.75 | 5.64 | uncharacterized protein LOC100834985 [*Brachypodium distachyon*] |
| comp84998_c0 | 8.77 | 9.21 | hypothetical protein TAA_ctg0091b.00060.1 [*Triticum aestivum*] |
| comp85212_c0 | 6.64 | 7.25 | predicted protein [*Hordeum vulgare*] |
| comp86133_c0 | 5.10 | 5.77 | hypothetical protein OsI_37755 [*Oryza sativa*] |
| comp86239_c0 | 6.23 | 5.52 |  |
| comp86361_c0 | 5.06 | 5.87 |  |
| comp92139_c0 | 5.44 | 6.47 | predicted protein [*Hordeum vulgare*] |
| comp93310_c0 | 5.51 | 5.66 | Os11g0211800 [*Oryza sativa*] |
| comp93790_c0 | 5.43 | 5.54 | uncharacterized protein LOC100834985 [*Brachypodium distachyon*] |
| comp95161_c0 | 6.28 | 6.37 | predicted protein [*Hordeum vulgare*] |
| comp95861_c1 | 6.36 | 7.20 | unknown protein [*Oryza sativa*] |
| comp96339_c0 | 7.03 | 7.62 |  |
| comp97165_c0 | 7.27 | 8.15 |  |
| comp97806_c0 | 5.17 | 6.03 | hypothetical protein ZEAMMB73_617483 [*Zea mays*] |
| comp98995_c0 | 5.82 | 5.37 | predicted protein [*Hordeum vulgare*] |
| Down-regulated genes |  |  |  |
| comp100013_c0 | -5.34 | -6.41 | predicted protein [*Hordeum vulgare*] |
| comp100368_c0 | -5.20 | -5.41 | uncharacterized protein LOC100841570 [*Brachypodium distachyon*] |
| comp100485_c1 | -5.55 | -5.78 | predicted protein [*Hordeum vulgare*] |
| comp100658_c0 | -5.25 | -7.12 | predicted protein [*Hordeum vulgare*] |
| comp101546_c0 | -5.28 | -5.54 | predicted protein [*Hordeum vulgare*] |
| comp101676_c0 | -5.13 | -7.15 | ABC transporter B family member 11-like [*Brachypodium distachyon*] |
| comp101831_c0 | -5.08 | -5.41 | predicted protein [*Hordeum vulgare*] |
| comp102345_c1 | -5.91 | -7.22 | probable indole-3-acetic acid-amido synthetase GH3.2-like [*Brachypodium distachyon*] |
| comp102601_c0 | -6.09 | -5.81 |  |
| comp102644_c0 | -5.04 | -10.91 | predicted protein [*Hordeum vulgare*] |
| comp102674_c0 | -5.17 | -6.62 | uncharacterized protein LOC100829724 [*Brachypodium distachyon*] |
| comp103580_c0 | -5.35 | -10.03 | predicted protein [*Hordeum vulgare*] |
| comp103741_c0 | -5.09 | -5.32 | hypothetical protein SORBIDRAFT_07g000920 [*Sorghum bicolor*] |
| comp103869_c0 | -5.39 | -8.87 |  |
| comp104048_c0 | -7.08 | -8.14 | predicted protein [*Hordeum vulgare*] |
| comp104296_c1 | -5.39 | -10.38 |  |
| comp104526_c0 | -6.03 | -6.76 |  |
| comp104554_c0 | -6.24 | -7.30 | predicted protein [*Hordeum vulgare*] |
| comp104646_c0 | -8.40 | -10.04 |  |
| comp104868_c0 | -5.85 | -9.99 | predicted protein [*Hordeum vulgare*] |
| comp105103_c0 | -5.69 | -7.53 | hypothetical protein SORBIDRAFT_09g018320 [*Sorghum bicolor*] |
| comp105888_c0 | -6.40 | -8.91 | uncharacterized protein LOC100840382 [*Brachypodium distachyon*] |
| comp106200_c0 | -5.04 | -6.91 | predicted protein [*Hordeum vulgare*] |
| comp106398_c0 | -5.76 | -7.58 | chymotrypsin-like protease [*Helicoverpa armigera*] |
| comp106673_c0 | -6.60 | -5.20 | putative invertase inhibitor-like [*Brachypodium distachyon*] |
| comp106812_c0 | -5.57 | -5.03 | predicted protein [*Hordeum vulgare*] |
| comp106919_c0 | -5.72 | -7.65 | CASP-like protein 1 [*Triticum aestivum*] |
| comp106919_c1 | -6.62 | -9.94 | CASP-like protein 1 [*Triticum aestivum*] |
| comp107023_c0 | -6.71 | -5.76 | group 11 grass pollen allergen F7-6 [*Secale cereale* x *Triticum durum*] |
| comp107500_c0 | -6.22 | -9.34 | predicted protein [*Hordeum vulgare*] |
| comp107536_c0 | -5.53 | -5.20 |  |
| comp107562_c0 | -5.16 | -5.05 | predicted protein [*Hordeum vulgare*] |
| comp107585_c0 | -5.24 | -9.84 | bidirectional sugar transporter SWEET6b-like [*Brachypodium distachyon*] |
| comp107636_c0 | -5.58 | -7.62 |  |
| comp107733_c0 | -5.61 | -8.80 | silicon transporter protein [*Triticum aestivum*] |
| comp107737_c0 | -5.33 | -7.36 | predicted protein [*Hordeum vulgare*] |
| comp107828_c0 | -5.51 | -10.48 | hypothetical protein SORBIDRAFT_09g025570 [*Sorghum bicolor*] |
| comp108406_c0 | -5.28 | -10.17 | polyphenol oxidase [*Triticum aestivum*] |
| comp108827_c0 | -6.77 | -9.44 | hypothetical protein OsI_24583 [*Oryza sativa*] |
| comp108842_c0 | -6.02 | -8.99 | predicted protein [*Hordeum vulgare*] |
| comp109056_c0 | -5.57 | -5.31 |  |
| comp109302_c0 | -5.92 | -8.63 | predicted protein [*Hordeum vulgare*] |
| comp109601_c0 | -5.04 | -6.41 | predicted protein [*Hordeum vulgare*] |
| comp109741_c0 | -5.76 | -5.86 | lipid transfer protein [*Triticum aestivum*] |
| comp109925_c0 | -5.63 | -8.17 | predicted protein [*Hordeum vulgare*] |
| comp110199_c0 | -5.43 | -7.01 | predicted protein [*Hordeum vulgare*] |
| comp110471_c0 | -5.38 | -9.18 | predicted protein [*Hordeum vulgare*] |
| comp110800_c0 | -5.87 | -5.04 | predicted protein [*Hordeum vulgare*] |
| comp110810_c0 | -5.30 | -5.79 | predicted protein [*Hordeum vulgare*] |
| comp111026_c0 | -6.50 | -6.34 | hypothetical protein [*Oryza sativa*] |
| comp111051_c2 | -6.16 | -5.34 | predicted protein [*Hordeum vulgare*] |
| comp111285_c0 | -5.72 | -7.80 | predicted protein [*Hordeum vulgare*] |
| comp111424_c1 | -6.00 | -9.73 | uncharacterized protein, partial [*Phleum pratense*] |
| comp111548_c0 | -5.00 | -10.40 | flavonoid 7-O-methyltransferase [*Hordeum vulgare*] |
| comp111760_c3 | -5.22 | -5.22 | predicted protein [*Hordeum vulgare*] |
| comp111939_c2 | -5.94 | -12.38 | hypothetical protein ZEAMMB73_870664 [*Zea mays*] |
| comp112020_c0 | -5.15 | -7.01 | naringenin,2-oxoglutarate 3-dioxygenase-like [*Brachypodium distachyon*] |
| comp112453_c0 | -5.06 | -8.64 | predicted protein [*Hordeum vulgare*] |
| comp112524_c0 | -6.12 | -6.55 | predicted protein [*Hordeum vulgare*] |
| comp112668_c1 | -7.57 | -9.35 | putative aldehyde decarbonylase enzyme CER1 [*Hordeum vulgare*] |
| comp113082_c0 | -5.39 | -6.71 | predicted protein [*Hordeum vulgare*] |
| comp113361_c0 | -5.64 | -10.02 | predicted protein [*Hordeum vulgare*] |
| comp113840_c0 | -6.30 | -11.52 | uncharacterized protein LOC100827428 [*Brachypodium distachyon*] |
| comp114079_c0 | -6.93 | -10.03 | predicted protein [*Hordeum vulgare*] |
| comp114094_c0 | -6.75 | -7.25 | predicted protein [*Hordeum vulgare*] |
| comp114326_c0 | -5.28 | -8.45 | hypothetical protein OsI_04209 [*Oryza sativa*] |
| comp114381_c0 | -5.10 | -5.55 | predicted protein [*Hordeum vulgare*] |
| comp114721_c0 | -5.12 | -5.78 | predicted protein [*Hordeum vulgare*] |
| comp114948_c0 | -5.39 | -6.62 | hypothetical protein ZEAMMB73_828118 [*Zea mays*] |
| comp114987_c0 | -5.83 | -11.10 | uncharacterized protein LOC100832503 [*Brachypodium distachyon*] |
| comp115018_c1 | -5.96 | -6.07 | predicted protein [*Hordeum vulgare*] |
| comp115045_c1 | -5.91 | -11.67 | sugar transport protein 1-like [*Brachypodium distachyon*] |
| comp116065_c1 | -5.02 | -5.93 | predicted protein [*Hordeum vulgare*] |
| comp116379_c0 | -6.51 | -5.35 | fasciclin-like protein FLA34 [*Triticum aestivum*] |
| comp116532_c0 | -5.25 | -5.81 | NADP-dependent glyceraldehyde-3-phosphate dehydrogenase [*Triticum aestivum*] |
| comp117315_c0 | -5.25 | -6.36 | predicted protein [*Hordeum vulgare*] |
| comp118336_c0 | -5.73 | -5.63 | cell wall invertase [*Triticum aestivum*] |
| comp118609_c0 | -6.25 | -7.90 | predicted protein [*Hordeum vulgare*] |
| comp118751_c0 | -5.00 | -8.20 | uncharacterized protein LOC100840940 [*Brachypodium distachyon*] |
| comp118917_c0 | -7.54 | -10.43 | predicted protein [*Hordeum vulgare*] |
| comp119476_c0 | -5.41 | -6.88 | predicted protein [*Hordeum vulgare*] |
| comp119714_c0 | -6.20 | -9.76 | ankyrin-like protein [*Oryza sativa*] |
| comp119820_c0 | -5.26 | -8.81 | Aspartic proteinase precursor, putative [*Ricinus communis*] |
| comp119854_c0 | -5.13 | -12.01 | uncharacterized protein LOC100837909 [*Brachypodium distachyon*] |
| comp119870_c1 | -5.11 | -7.34 | predicted protein [*Hordeum vulgare*] |
| comp120172_c1 | -5.34 | -8.89 | predicted protein [*Hordeum vulgare*] |
| comp121380_c0 | -7.44 | -7.24 | predicted protein [*Hordeum vulgare*] |
| comp121453_c0 | -5.01 | -11.17 | monosaccharide-sensing protein 3-like [*Brachypodium distachyon*] |
| comp121488_c1 | -5.86 | -5.25 | ACC oxidase [*Hordeum vulgare*] |
| comp122918_c0 | -5.15 | -5.34 | BEL1-type homeodomain protein WBLH1 [*Triticum aestivum*] |
| comp123112_c0 | -5.51 | -7.58 | uncharacterized protein LOC100830416 [*Brachypodium distachyon*] |
| comp123336_c0 | -5.11 | -10.19 | hypothetical protein OsI_13514 [*Oryza sativa*] |
| comp123515_c0 | -7.66 | -10.12 |  |
| comp124542_c0 | -7.18 | -9.43 |  |
| comp126731_c0 | -6.46 | -8.59 | chemocyanin, partial [*Zea mays*] |
| comp127876_c0 | -5.67 | -9.75 | GDSL esterase/lipase At5g03610-like [*Brachypodium distachyon*] |
| comp128379_c0 | -5.44 | -10.99 | Os11g0582500 [*Oryza sativa*] |
| comp131846_c0 | -6.37 | -5.83 |  |
| comp134243_c0 | -7.44 | -11.61 | predicted protein [*Hordeum vulgare*] |
| comp139174_c0 | -5.32 | -5.46 | Putative retroelement [*Oryza sativa*] |
| comp151384_c0 | -5.40 | -6.53 | hypothetical protein SORBIDRAFT_02g024990 [*Sorghum bicolor*] |
| comp24712_c0 | -6.94 | -7.07 |  |
| comp31698_c0 | -5.79 | -8.30 |  |
| comp72047_c0 | -7.08 | -12.63 |  |
| comp78647_c0 | -5.63 | -5.76 |  |
| comp78882_c0 | -6.49 | -7.32 |  |
| comp86141_c0 | -6.11 | -7.46 | predicted protein [*Hordeum vulgare*] |
| comp86311_c0 | -5.68 | -8.55 | protein pelota-like [*Brachypodium distachyon*] |
| comp91418_c0 | -5.44 | -5.24 |  |
| comp93539_c0 | -5.04 | -7.29 | uncharacterized protein LOC100833110 [*Brachypodium distachyon*] |
| comp94850_c0 | -5.39 | -5.70 | cytochrome P450 89A2-like [*Brachypodium distachyon*] |
| comp94857_c0 | -5.34 | -5.78 | uncharacterized protein LOC100275644 [*Zea mays*] |
| comp96190_c0 | -6.19 | -10.69 | RNase S-like protein precursor [*Hordeum vulgare*] |
| comp96975_c0 | -7.97 | -8.94 | dehydrin [*Hordeum vulgare*] |
| comp97007_c0 | -5.38 | -5.52 | predicted protein [*Hordeum vulgare*] |
| comp97025_c0 | -5.45 | -5.28 | predicted protein [*Hordeum vulgare*] |
| comp97346_c0 | -8.69 | -6.11 |  |
| comp97653_c0 | -5.07 | -5.12 |  |
| comp98886_c0 | -5.57 | -8.95 |  |
| comp99455_c0 | -5.01 | -6.53 | protein 1C,chlorophyll binding [*Triticum aestivum*] |
| comp99464_c0 | -6.71 | -9.36 | PREDICTED: profilin-2/4-like [*Brachypodium distachyon*] |
| comp99522_c0 | -6.51 | -5.73 | phosphate transporter HvPT7 [*Hordeum vulgare*] |
| comp99613_c0 | -5.06 | -6.76 | elongation factor 2-like [*Brachypodium distachyon*] |
