# Supplementary material for: Pistillody mutant reveals key insights into stamen and pistil development in wheat (Triticum aestivum L.)
Source: BMC Genomics. 2015 Mar 19;16(1):211. doi: 10.1186/s12864-015-1453-0 (PMC4369888; doi:10.1186/s12864-015-1453-0)
Supplement: Additional file 4: Table S3. — The primers designed for qRT-PCR. [file 12864_2015_1453_MOESM4_ESM.docx]

Table S3 The primers designed for qRT-PCR

| Gene | Primer Sequence | | Products size (bp) |
| --- | --- | --- | --- |
| comp79245_c0 | Forward | 5＇TCGTCTTGGGAATGGGAGG 3＇ | 86bp |
|  | Reverse | 5＇TTAACCGGCTACGTGTAATGCT 3＇ |  |
| comp84998_c0 | Forward | 5＇CAGCCAGATCCAGATACCACA 3＇ | 136bp |
|  | Reverse | 5＇ AGACAGAGGCAGGAGGAAACTT3＇ |  |
| comp118917_c0 | Forward | 5＇AGCAAGAAACAACCCCAATCT 3＇ | 97bp |
|  | Reverse | 5＇GTCCAATCCGTCGTCTGAAA 3＇ |  |
| comp41682_c0 | Forward | 5＇TGGCACGGTATGTACTGCGTAT 3＇ | 140bp |
|  | Reverse | 5＇CGAAGCGGCGGGAGATTAT 3＇ |  |
| comp116835_c0 | Forward | 5＇CCCAACCGTGTAAAACACCTC 3＇ | 88bp |
|  | Reverse | 5＇CCTCGTCTACCCCATGAACC 3＇ |  |
| comp103277_c0 | Forward | 5＇TGGGTATCGGAAGGGTGGA 3＇ | 133bp |
|  | Reverse | 5＇CGAAAGACGTACTGGTCAGTGTAA 3＇ |  |
| comp72047_c0 | Forward | 5＇CGCTAGTTTCCAGGGAAGACC 3＇ | 143bp |
|  | Reverse | 5＇CCTCGTCTACCCCATGAACC 3＇ |  |
| comp126922_c0 | Forward | 5＇TCAGTTCCTGTCCGCTGTCTT 3＇ | 90bp |
|  | Reverse | 5＇GAGGCGACGGTGCAGTTTT 3＇ |  |
| comp124542_c0 | Forward | 5＇CGAAACAACATGCAGCACTCT 3＇ | 88bp |
|  | Reverse | 5＇CACCGCTCATCGTTACTTGG 3＇ |  |
| comp104646_c0 | Forward | 5＇ATTTTCAAGGGAATAGTTCGTCTC 3＇ | 146bp |
|  | Reverse | 5＇TCGCTTCACGGTACAAGGC 3＇ |  |
| comp112668_c1 | Forward | 5＇AGAAGGTCTGGATGATGCTGAG 3＇ | 80 bp |
|  | Reverse | 5＇TCTTTGGCGGGAACTGTGA 3＇ |  |
| comp109228_c0 | Forward | 5＇CGGAACAACATTTTCAGCGTAG 3＇ | 104 bp |
|  | Reverse | 5＇GGGCAACCGAGGAACCAAT 3＇ |  |
| comp125818_c0 | Forward | 5＇AAGCTAATCTAGCCATGAGTCCTT 3＇ | 130bp |
|  | Reverse | 5＇TTGTCCGTGATAAAGTGGTCG 3＇ |  |
| comp123515_c0 | Forward | 5＇CGGTCTTTCTCACCCGTCAC 3＇ | 103bp |
|  | Reverse | 5＇GCGTCTTGTTCCATCCATCC 3＇ |  |
| comp121380_c0 | Forward | 5＇GGTAGAGTTGCCGTTGTCGC 3＇ | 128 bp |
|  | Reverse | 5＇TGAAATACTGGAGCACTGTGAATG3＇ |  |
| comp134243_c0 | Forward | 5＇TCACGGAGTATGGTGGCAAT3＇ | 132bp |
|  | Reverse | 5＇ACCTTTCGAGGAGAAGTTCGG 3＇ |  |
| comp97346_c0 | Forward | 5＇ACAAACGAAGCTGCTCCATG 3＇ | 93bp |
|  | Reverse | 5＇CAACAATATCACCACCAAAACG 3＇ |  |
| comp108901_c0 | Forward | 5＇TGAGCATGGGCATGGACTC 3＇ | 121bp |
|  | Reverse | 5＇AACGGAGAAGCGGCTATCG 3＇ |  |
| comp108229_c0 | Forward | 5＇CACTGCCTGGTGGTGGTAGTA 3＇ | 134bp |
|  | Reverse | 5＇TGTATCTCCGAGCCAAGGTG 3＇ |  |
| comp110066_c0 | Forward | 5＇ACCCATAAAGTTGCCCGAGAC 3＇ | 105bp |
|  | Reverse | 5＇TTTCCCTTGAGACGCACTACAC 3＇ |  |
| comp110106_c0 | Forward | 5＇GCAACGGAACCTAGTATGTATCGG 3＇ | 99bp |
|  | Reverse | 5＇ACACCAGCACAGGCACCAA 3＇ |  |
| comp112313_c0 | Forward | 5＇GCAGTTGCAGTGGACGTAGC 3＇ | 134bp |
|  | Reverse | 5＇GCAGACTCAGGGCATAGCG 3＇ |  |
| comp113007_c0 | Forward | 5＇CGTGCGAGGGAAGCG 3＇ | 106bp |
|  | Reverse | 5＇GACAAGGCAAGCCAAGATG 3＇ |  |
| comp112668_c0 | Forward | 5＇TTCAGAAGTCCAAGGCTAACA 3＇ | 91bp |
|  | Reverse | 5＇AGGAGAAGGAGGCAATCAA 3＇ |  |
| comp122918_c0 | Forward | 5＇AGAGGGAGACAAGCGGAGACAG 3＇ | 102bp |
|  | Reverse | 5＇CAACCTAAGATGAGGAGGAGCACA 3＇ |  |
| Actin | Forward | 5＇ACGCTTCCTCATGCTATCCTT C 3＇ | 121bp |
|  | Reverse | 5＇ATGTCTCTGACA ATTTCCCGC T 3＇ |  |
| Ubiq | Forward | 5＇AAGGCGAAGATCCAGGACAAG 3＇ | 107bp |
|  | Reverse | 5＇TGGATGTTGTAGTCCGCCAAG 3＇ |  |
